# Bile and urine peptide marker profiles: access keys to molecular pathways and biological processes in cholangiocarcinoma

**DOI:** 10.1186/s12929-019-0599-5

**Published:** 2020-01-03

**Authors:** Torsten Voigtländer, Jochen Metzger, Holger Husi, Martha M. Kirstein, Martin Pejchinovski, Agnieszka Latosinska, Maria Frantzi, William Mullen, Thorsten Book, Harald Mischak, Michael P. Manns

**Affiliations:** 1 Department of Gastroenterology, Hepatology and Endocrinology, Hannover Medical School, Hannover, Germany; 2 Mosaiques diagnostics GmbH, Rotenburger Straße 20, 30659 Hannover, Germany; 3 Division of Biomedical Sciences, Centre for Health Science, University of the Highlands and Islands, Inverness, UK; 4 Institute of Cardiovascular and Medical Sciences, Glasgow, UK

**Keywords:** Cholangiocarcinoma, Biomarkers, Biomolecular pathways, Proteomics

## Abstract

**Background:**

Detection of cholangiocarcinoma (CCA) remains a diagnostic challenge. We established diagnostic peptide biomarkers in bile and urine based on capillary electrophoresis coupled to mass spectrometry (CE-MS) to detect both local and systemic changes during CCA progression. In a prospective cohort study we recently demonstrated that combined bile and urine proteome analysis could further improve diagnostic accuracy of CCA diagnosis in patients with unknown biliary strictures. As a continuation of these investigations, the aim of the present study was to investigate the pathophysiological mechanisms behind the molecular determinants reflected by bile and urine peptide biomarkers.

**Methods:**

Protease mapping and gene ontology cluster analysis were performed for the previously defined CE-MS based biomarkers in bile and urine. For that purpose, bile and urine peptide profiles (from samples both collected at the date of endoscopy) were investigated from a representative cohort of patients with benign (*n* = 76) or CCA-associated (*n* = 52) biliary strictures (verified during clinical follow-up). This was supplemented with a literature search for the association of the individual biomarkers included in the proteomic patterns with CCA or cancer progression.

**Results:**

For most of the peptide markers, association to CCA has been described in literature. Protease mapping revealed ADAMTS4 activity in cleavage of both bile and urine CCA peptide biomarkers. Furthermore, increased chymase activity in bile points to mast cell activation at the tumor site. Gene ontology cluster analysis indicates cellular response to chemical stimuli and stress response as local and extracellular matrix reorganization by tissue destruction and repair as systemic events. The analysis further supports that the mapped proteases are drivers of local and systemic events.

**Conclusions:**

The study supports connection of the CCA-associated peptide biomarkers to the molecular pathophysiology and indicates an involvement in epithelial-to-mesenchymal transition, generation of cancer-associated fibroblasts and activation of residual immune cells. Proteases, extracellular matrix components, inflammatory cytokines, proangiogenic, growth and vasoactive factors released from the tumor microenvironment are drivers of systemic early events during CCA progression.

## Background

Cholangiocarcinoma (CCA) is a rare but highly aggressive adenocarcinoma of the bile ducts accounting for 3% of all gastrointestinal cancers and 15% of all primary liver cancers worldwide [1]. CCA is diagnosed in 5000 new cases on average per year in the United States [2], which is translated to an annual incidence of 2–3 per 100,000 worldwide. Moreover, the incidence of CCA is still rising per year by approximately 3% [3].

Due to poor response to chemotherapy, early surgery is currently the only curative treatment option available. However, it is associated with 5–10% mortality rates and even with margin-free resection, 5-year survival rates only reach 20–40%. In 50–95% of cases, CCA is detected at a stage when it is too late for resection. In this case, prognosis is poor with median survival being 5 months. In 2018, CCA accounted for 165,087 of deaths worldwide according to the GLOBOCAN database (released in September 2018).

The detection of CCA remains a diagnostic challenge, particularly in patients with primary sclerosing cholangitis (PSC), a chronic cholestatic liver disease characterized by progressive inflammation and fibrosis of the bile ducts. These patients develop CCA in approximately 30% of all cases during the first 10 years of the disease [4]. A major clinical challenge is the discrimination of CCA from benign bile duct strictures, even more considering that CCA may develop quite early in the disease course. One approach to improve life-saving curative treatment (e.g. surgical liver resection or orthotopic liver transplantation), is to enable early detection of CCA with high accuracy. Currently, CCA is detected by imaging such as computed tomography, magnetic resonance imaging or endoscopic procedures with subsequent histological examination, with modest accuracy, at best with estimated sensitivity of 60% [5–8]. Two case/control studies were previously published using bile [9] or urine [10] as sample matrix and resulted in peptide biomarker models consisting of 22 biliary and 42-urinary peptides, respectively. Both studies indicate that proteomic analysis is a suitable method to detect CCA in patients with PSC based on peptide multi-marker models with classification accuracies above 80% [9, 10]. Recently, a case-control phase II study on 87 patients (36 CCA including 5 with CCA on top of PSC, 33 PSC and 18 other benign disorders) was initiated to elucidate the potential of a combined bile and urine proteomic analysis at the date of endoscopy for improved CCA diagnosis, resulting in an area under the curve (AUC) value of 0.96 [95% Confidence Interval (CI): 0.89 to 0.99] in receiver operating characteristics (ROC) analysis [11]. In a prospective evaluation on 45 subsequently collected patients with a six months clinical follow-up, combined bile and urine proteome analysis demonstrated an accuracy for CCA detection of 92% and a negative predictive value of 96% [11]. Most importantly, only in 4 out of the 16 patients with CCA included in the prospective study the tumor could subsequently be detected by imaging techniques.

For classification of patient samples by CE-MS, the normalized retention time in CE and MS-detected molecular mass are required for characterization of each specific peptide marker. However, additional determination of the amino acid sequence provides the link to the parental protein and as such a connection to the disease pathophysiological processes. Most of the peptides included in the bile and urine proteome model could be identified by peptide sequencing [11]. Peptides with differential excretion levels in bile of patients with CCA compared to those with benign strictures, were fragments from the hemoglobin subunits α (HBA1) and β (HBB), the inter-α trypsin inhibitor heavy chains H2 (ITIH2) and H4 (ITIH4), cytoplasmic actin 1 (ACTB) and serum albumin (ALB). In bile, a fragment of the 14–3-3 ζ/δ protein (YWHAZ) was observed, which is known to be released by trypsin cleavage [12]. In urine, peptides derived from uromodulin (UMOD), CD99 antigen (CD99), Na^+^/K^+^ ATPase subunit γ (FXYD2) and membrane associated progesterone receptor component 1 (PGMRC1) were decreased [11]. Specific collagen fragments were deregulated, indicative of changes in the extracellular matrix environment and proteolytic turnover. Based on these findings, a hypothesis was generated that the bile peptide marker pattern may depict local changes and the urinary peptide marker pattern may reflect systemic changes of CCA progression, raising the question how local and systemic changes are connected to each other and are linked to the pathophysiology of CCA.

The aim of the present study was to investigate potential links of the identified changes in bile and urine peptides with pathophysiological mechanisms for CCA progression, specifically in distinction to benign biliary strictures. This investigation was based on all 16 biliary and 30 urinary peptide markers included in the bile and urinary peptide marker models described above for which the amino acid sequence could be determined. Using this set of CCA peptide markers, proteases that are associated with the transition from local biliary to systemic urinary CCA manifestations were defined through in silico protease mapping based on the N- and C-terminal ends of the bile and urinary peptide markers. Moreover, interactions between parental proteins and proteases involved in the generation of the peptides, along with their biological relevance were investigated by gene ontology cluster analysis. The approach might uncover key information for a better understanding of the disease mechanisms driving this rapidly growing and fast metastasizing biliary tract tumor and may further open new avenues for antitumor therapy.

## Methods

### Patients and samples

In this study, paired bile and urine proteome profiles were analyzed from a total of 128 patients collected at the same date during endoscopy of the biliary tract including patients recruited during previous clinical studies (*n* = 123) [9–11] and five additional patients donating bile and urine samples. All patients were admitted to the gastroenterology department of the Medical School Hannover, Germany, between January 2008 and April 2018 for diagnosis of biliary diseases or monitoring of disease progression mainly in connection with an endoscopy of the biliary tract. The diagnosis of benign or malignant strictures was based on cholangiographic findings and histological examinations with a clinical follow-up of at least one year. Special care was given to ensure that none of the patients with benign biliary strictures showed clinical or imaging signs of CCA during one year of clinical follow-up. The patient cohort consists of 33 patients with CCA findings without prior PSC, 19 patients with CCA concomitant to PSC (CCA on top of PSC), 57 patients with PSC and 19 patients with other benign biliary disorders, mainly choledocholithiasis (*n* = 9), but also chronic pancreatitis (*n* = 3), secondary sclerosing cholangitis (*n* = 3) and common bile duct dilatation (*n* = 3). Demographic and clinical data of the 128 patients is presented in Additional file 1: Table S1. Exclusion criteria were set to a minimum excluding only patients below 18 years of age or who underwent liver transplantation. All patients gave their written informed consent. Proteomic analyses were approved by the local ethical committee of Hannover Medical School.

### CE-MS analysis and proteomic data processing

Patient’s bile and urinary peptide profiles are derived from previous CE-MS analyses [9–11]. Briefly, CE-MS instrumentation consists of a P/ACE MDQ capillary electrophoresis system (Beckman Coulter, Fullerton, USA) on-line coupled to a Micro-TOF MS (Bruker Daltonic, Bremen, Germany) as the basis for continuous and seamless sample analysis. Other specific adaptations for automated and fast (60 min for one analysis cycle) sample analysis have been adopted, such as grounding of the ESI sprayer (Agilent Technologies, Palo Alto, USA), setting the ion spray interface potential between − 4.0 and − 4.5 kV and MS data acquisition automatically controlled by the CE via contact-close-relays. Spectra are accumulated every 3 s over a mass-to-charge ratio ranging from 350 to 3000. Details on accuracy, precision, selectivity, sensitivity, reproducibility, and stability of the CE-MS method are reported elsewhere [13, 14].

Raw proteomic data processing was carried out by an in-house software MosaiquesVisu [15], which deconvolutes mass spectral ion peaks at different charge states into real peptide masses by their isotopic distribution and conjugated masses. After this step, a list of peptides in the sample is generated where each peptide entry is characterized by its molecular mass, CE-migration time, and ion signal intensity. For inter-comparability across measurements, each of these parameters is normalized by global and local linear regression using internal standard peptides of high abundance, low ion signal variability and FT-ICR MS-determined exact molecular mass [16]. All detected peptides are deposited, matched, and annotated in a Microsoft SQL database for further analysis and comparison of multiple samples. Peptides are considered identical within different samples, when mass deviation is lower than 50 ppm for small peptides or 75 ppm for larger peptides and proteins. Due to analyte diffusion effects, CE peak widths increase with CE migration time which is compensated by linearly increasing the accepted values for the percentage of deviation from 2 to 5% in the relevant CE time range from 19 to 45 min after sample injection. After calibration, deviation of migration time is controlled to be less than 0.45 min.

### In silico protease mapping

In silico protease mapping to the amino acid sequence of peptide markers included in the bile and urinary proteomic models for CCA was performed as described previously [17]. Briefly, on the basis of the N- and C-terminal amino acid sequence motifs of the bile and urinary CCA peptide markers and the protease specificity weight matrices provided by the MEROPS database (http://merops.sanger.ac.uk/index.shtml) associated proteases were predicted by Proteasix [18]. Besides these positive associations, a list of random octapeptide sequences was mapped by Proteasix to determine the specificity of the prediction and to assess the false discovery rate. Only proteases that have a positive predictive value (% of true positive / true positive + false positive) higher than 65% and associated with at least four cleavage sites were kept for further analysis. Protease activity was subsequently assessed in the patient’s CE-MS peptide profiles based on the average of associated peptide intensities in the CCA cases compared to the PSC or other benign strictured control subjects. Statistical significance was determined using the Mann Whitney U Test from the MedCalc® 12.7.5.0 statistical software package (Mariakerke, Belgium).

### Gene ontology cluster analysis

Modulated and inferred molecules associated with bile or urine CCA expression were subjected to Gene Ontology (GO) analysis using Cytoscape v3.5.0, ClueGO v 2.5.3, CluePedia v1.5.3, and the GO biological process definition file (download date 30.1.2019). Settings and thresholds applied were defined as human, Uniprot identifiers, analysis p-term values ≤0.05 and kappa score threshold of 0.45. The statistical significance of the findings was assessed using an enrichment/depletion two-sided hypergeometric test including the Bonferroni step-down correction method for multiple testing.

### Tissue transcriptomics data evaluation

Tissue transcriptomics data were retrieved from the NCBI Gene Expression Omnibus (GEO) database available at https://www.ncbi.nlm.nih.gov/geo/ [19, 20]. This includes expression profiling data by array from: i) tumor and paired non-malignant tissue of CCA patients (GEO accession: GSE76297) [21], ii) CCA tumor and non-malignant matched surrounding livers as well as normal bile ducts tissue (GSE26566) [22], iii) tumor and paired non-malignant liver tissues from intrahepatic CCA patients as well as benign liver lesions tissue from focal nodular hyperplasia cases (GSE32879) [23], and iv) laser capture micro-dissected fibrous tissue from tumor and non-malignant areas from intrahepatic CCA patients (GSE45001) [24]. Data were analyzed with GEO2R (https://www.ncbi.nlm.nih.gov/geo/geo2r/, [20]), an online tool, allowing for the comparison of samples within GEO Series. The analysis was conducted using default settings, with adjustments of *p*-values based on the Benjamini & Hochberg method [25].

As a second transcriptomics data source the “Pan-Cancer Analysis of Whole Genomes” project RNA-Seq mRNA repository available at https://www.ebi.ac.uk/ was used. Gene expression levels as “transcripts per million” (TPM) for the targeted proteins were compared in form of box-and-whisker plot representations for all currently (October 2019) available 18 CCA, 18 CCA-adjacent, 100 HCC, 53 HCC-adjacent and 35 normal liver tissue replicates.

### Literature search strategy

A systematic literature review was performed for the proteins from which the bile and urine peptide markers are derived through the PubMed database on May 13th, 2019 using the name of the protein in combination with cholangiocarcinoma, cholangiocellular carcinoma or bile duct cancer to retrieve records for their relation to CCA pathology and in a second search with cancer for their relation to cancer pathology in general.

### Overall survival and logistic regression analysis

Overall survival was analyzed using Kaplan-Meier methodology. In survival analysis, a log-rank test was performed to compare patients with a combined bile and urine proteomic positive test result versus those with a negative test result. Binomial logistic regression was performed to determine the relationship between classification by the combined bile and urine proteomic test with demographic and clinical data in predicting patient’s death. For both statistical analyses the MedCalc® 12.7.5.0 software package was used.

## Results

### CCA diagnosis based on bile and urine proteome analysis

Based on the proteome analysis, a patient sample is assigned to the case or control group according to the degree of similarity in its individual marker profile to the prototypical CCA peptide marker signature with the later defined during the establishment of the peptide marker model. The SVM-based classification model in this respect transforms the high-dimensional parameter space composed of the log-transformed ion signal distributions of all included peptides to numerical membership probability values. Membership probability in this respect is quantified by the Euclidean distance of the data point to the maximal margin of a separation hyperplane between the cases and controls that were used to train the model in a multidimensional space constructed by the peptide classifiers. Membership probability therefore expresses the degree of similarity of a patients’ peptide profile to the one that was identified to be specific for the disease in the discovery phase on clinically well-defined patients. Bile proteome analysis is indicative for CCA if the membership probability value is greater than 0.08. In the case of urine proteome analysis the threshold for CCA test positivity is − 0.89. Same as the threshold, the range of membership probability values is a characteristic feature of the SVM peptide marker pattern and ranges from − 2.5 to 2.5 in the case of bile and − 3.5 to 3.5 in the case of urine proteome analysis.

To combine both proteomic analyses into one diagnostic test for patients at the date of endoscopy of the bile ducts, logistic regression analysis was subsequently performed using clinical diagnosis (absence/presence) of CCA as the dependent binary variable and the membership probability values of bile and urine proteome analysis as independent variables. Based on the estimated correlation coefficients of 1.8325 for BPA and 2.6363 for UPA the regression equation reads as follows: BPA/UPA match = 1.6128 + 1.8325 * BPA score + 2.6363 * UPA score. As reported by Voigtländer et al. [11], combined bile and urine proteome analysis resulted in improved accuracy of CCA diagnosis in prospective evaluation if the predetermined threshold of − 0.52 for a positive CCA test result was applied.

### Impact of a positive proteomic test on overall patient survival

**Fig. 1:**
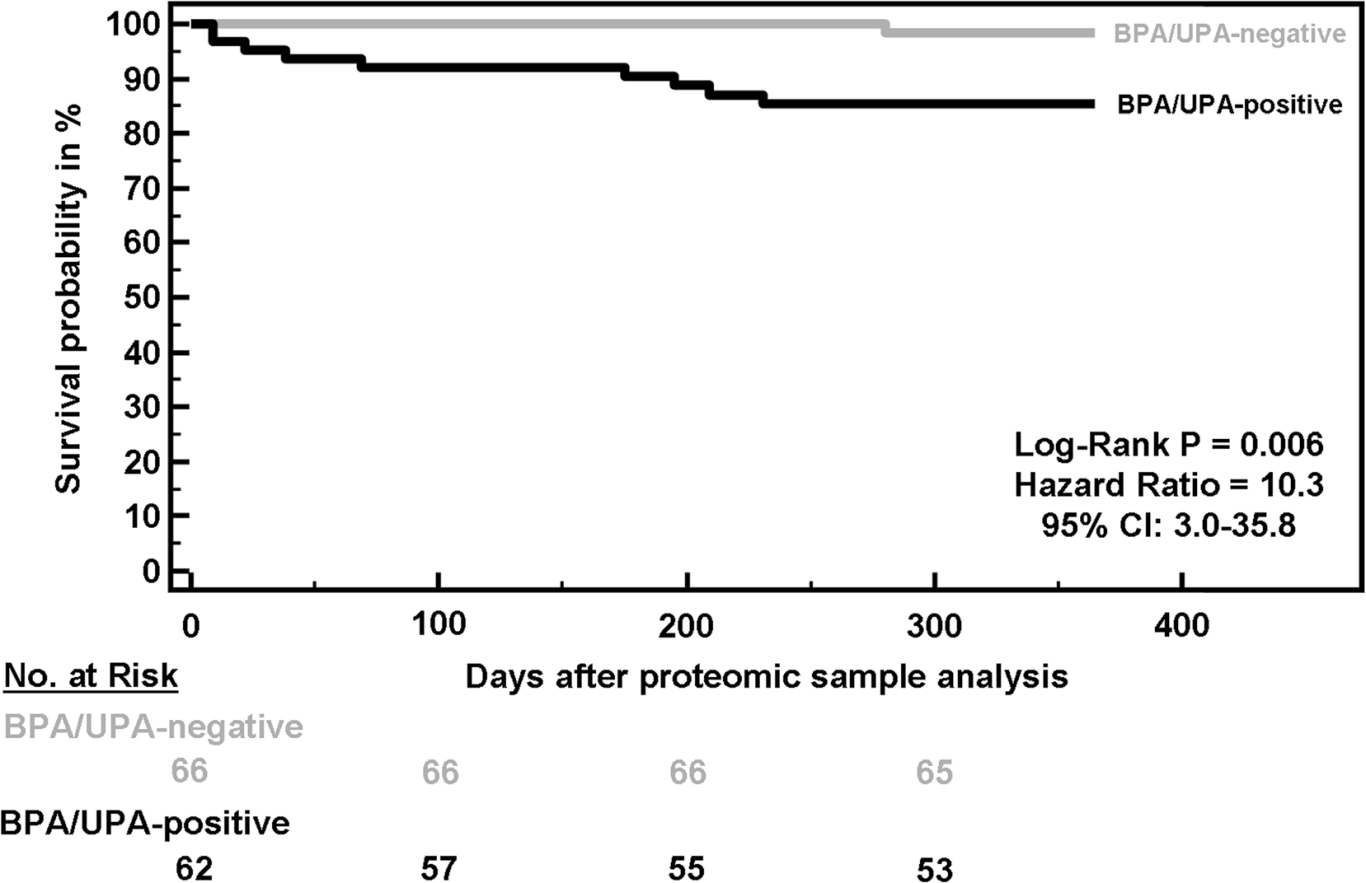
Overall survival of patients with a positive or negative test result in combined bile (BPA) and urine (UPA) proteome analysis during a 1-year follow-up starting from the date of endoscopy at which bile and urine were obtained from the patients

Based on the assumption that CCA, in contrast to PSC, is associated with poor prognosis, we hypothesized that the high CCA classification accuracy by combined bile and urine proteome analysis enables detecting significant differences in the overall survival probabilities between patients with a negative and a positive proteomic test result. Based on the follow-up data that are available, and as presented in Fig. 1, patients with a proteomic test positive for CCA have a 10.3-fold increased risk of death (95% CI: 2.98–35.8, *p* = 0.006) during a 1-year follow-up compared to patients with a negative proteomic test. In absolute numbers, nine of the 62 patients (14.5%) with a positive proteomic test result had died, in contrast to only one of the 66 patients (1.5%) with a negative proteomic test result. All nine patients who died within the first year of follow-up in the positive proteomic test group were clinically diagnosed as having CCA. Death in this group was mainly caused by combinations of gastrointestinal bleeding, intestinal ischemia, multi organ failure, pulmonary embolism and acute kidney injury. This not only demonstrates the tests’ high diagnostic accuracy, but also justifies the classification threshold of > − 0.5 for CCA positivity as determined by statistical means in our previous study [11].

Multivariate logistic regression analysis was performed using death within one year after performing the combined bile and urine proteomic test as dependent binary variable and classification factors of bile and urine proteome analysis together with demographic and clinical parameters as independent variables. As shown in Table 1, the classification factor of combined bile and urine proteome analysis is the only significant predictor of mortality in this patient cohort.

**Table 1:** Multiparametric logistic regression analysis to express the relationship of classification by the combined BPA/UPA proteomic test with demographic and clinical variables for the prediction of overall survival within the first year of clinical follow-up after proteomic analysis. The regression coefficient in the table expresses the amount of change in the logit function related to one unit change in the predictor

Independent variable	Regression coefficient	Standard error	Significance level *P*	Odds ratio (95% CI)
BPA/UPA match	0.5170	0.2240	0.02	1.68 (1.08–2.60)
Age (year)	0.0056	0.0351	0.87	1.01 (0.94–1.08)
Gender (female = 0, male = 1)	−0.1811	1.1347	0.87	0.83 (0.09–7.71)
Alkaline phosphatase (U/L)	−0.0117	0.0065	0.07	0.99 (0.98–1.00)
γ-Glutamyltransferase (U/L)	0.0017	0.0016	0.31	1.00 (1.00–1.00)
Bilirubin (μmol/L)	−0.0088	0.0089	0.33	0.99 (0.97–1.01)
Leucocyte count (N)	0.00026	0.00017	0.12	1.00 (1.00–1.00)
C-reactive protein (mg/L)	0.0134	0.0103	0.20	1.01 (0.99–1.03)
Alanine aminotransferase (U/L)	−0.0071	0.0159	0.66	0.99 (0.96–1.02)
Aspartate aminotransferase (U/L)	0.0202	0.0249	0.42	1.02 (0.97–1.07)
Carbohydrate antigen 19–9 (kU/L)	0.000046	0.000092	0.62	1.00 (1.00–1.00)

### Peptide marker profiles in bile and urine for benign and malignant strictures of the biliary tract

**Fig. 2:**
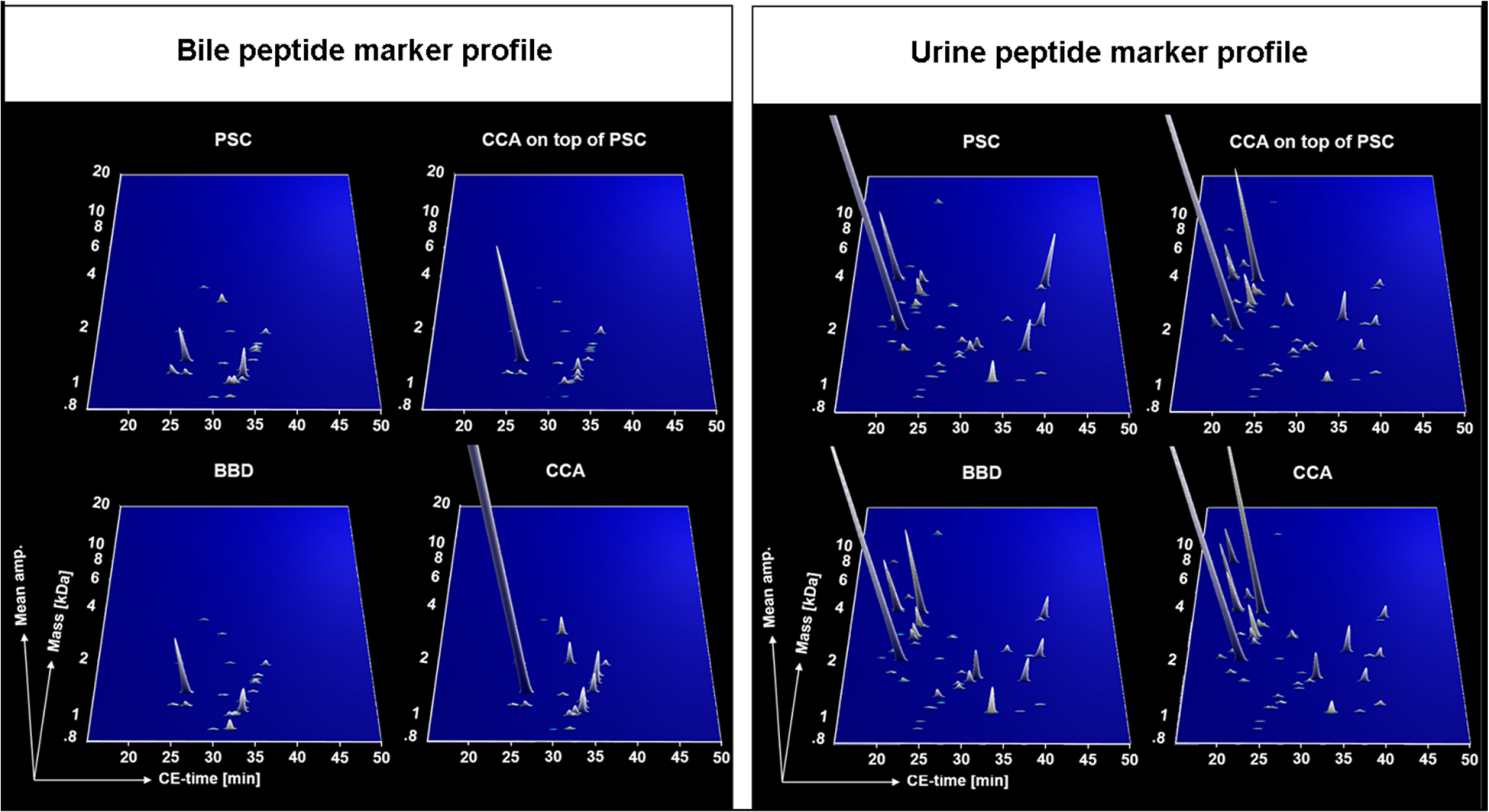
Group-specific CE-MS peptide marker profiles for primary sclerosing cholangitis (PSC, *n* = 57), benign biliary diseases (BBD, *n* = 19) other than PSC, cholangiocarcinoma (CCA, *n* = 33) alone or concomitant to PSC (CCA on top of PSC, *n* = 19) in bile (BPA) and urine (UPA) proteome analysis. The molecular mass on a logarithmic scale (0.8–20 kDa) is plotted against normalized capillary electrophoresis (CE) migration time (18–50 min). Mean signal intensities are encoded by peak height

The group-specific peptide marker profiles for bile (left panel of Fig. 2) and urine (right panel of Fig. 2) represent signatures of disease phenotypes at the proteomic level. The bile and urine peptide marker profiles presented in Fig. 2 indicate that patients with CCA concomitant to PSC include features of both pure PSC and pure CCA, resembling an intermediate state molecular profile. This is evident more in the bile than in the urine marker profile. The compiled bile marker profile of BBD’s other than PSC represents peptide marker expressions that are closely related to the respective CCA profiles than this is the case for PSC. This provides an explanation for the reduced, nevertheless significant ability of the bile peptide marker profile to discriminate BBD’s other than PSC, mostly choledocholithiasis, from the CCA cases.

### Protease prediction based on terminal amino acid motifs of peptides included in the bile and urine proteome-based peptide marker profiles

Peptides likely represent disease-associated changes after proteolysis [18]. We performed in silico protease mapping to the identified peptide markers independently from each other in the bile and urine proteomic models for on-site and peripheral proteolytic alterations. 15 protease candidates were identified in bile and 20 in urine fulfilling the sequence-based mapping criteria. Of these, three in each biofluid displayed significant differences in peptide distribution and intensities between CCA case and benign control samples. As presented in Table 2, the proteases ‘a disintegrin and metalloproteinase with thrombospondin motifs 4′ (ADAMTS4), chymase (CMA1) and kallikrein-4 (KLK4) were predicted based on the bile peptide markers to be significantly increased in CCA compared to benign strictures (*p* <  0.05). ADAMTS4 was also predicted with increased proteolytic activity when protease prediction was performed on urinary peptide markers. The same accounts for KLK4, however, in the case of the urinary peptides this did not reach statistical significance (*p* = 0.11). Whereas no protease was identified in bile with decreased activity, the proteases caspase-1 (CASP1) and kallikrein-6 (KLK6) were predicted based on the cleaved urine peptides of decreased activity in CCA in comparison to benign strictures. The predicted proteases based on cleavage site associations for the bile and urine CCA peptide markers are presented in Additional file 1: Table S2.

**Table 2:** Proteases predicted by in silico protease mapping to peptide markers included in the CCA-specific bile and urine proteomic models. Analysis was carried out for bile and urine peptide markers independent from each other. Differences in the predicted activities between the case and control groups are represented by the fold change between the ion signals of the protease associated peptide substrates in the case and control groups. Fold change calculations are based on the following principles: Ion signals for the CCA peptide markers are extracted from the normalized peptide lists of individual samples as described in the methods section. Protease prediction is performed on the set of CCA peptide markers for each individual patient. CCA peptide markers for which the N- or C-terminal amino acid sequence motif could be attributed to the same protease were integrated by their ion signals to calculate the proteolytic activity of that protease in one patient. In a final step, proteolytic activity of a protease is integrated over all patients of one group and group wise compared to generate the final list of differences in protease activities based on the peptides ion signal distributions in the case versus the control patient group. *P* values were calculated by the Mann Whitney U test. Abbreviations: *BBD* benign biliary disease, *CCA* cholangiocarcinoma, *PSC* primary sclerosing cholangitis

Protease	Peptide origin	Peptide substrate distribution [Avg. ion counts ± SD]		*P*
		CCA case group	PSC/other BBD control group	
ADAMTS4	Bile	670.33 ± 2100.31	95.35 ± 239.85	0.014
CMA1		353.85 ± 903.46	108.50 ± 225.54	0.0018
KLK4		407.48 ± 1309.74	68.33 ± 168.92	0.0072
ADAMTS4	Urine	429.51 ± 522.50	251.30 ± 434.86	0.006
CASP1		390.00 ± 627.39	537.32 ± 544.00	0.0007
KLK6		144.39 ± 189.94	289.47 ± 243.20	< 0.0001

### Functional gene ontology group-clustering of protein ancestors and proteases associated with the peptides included in the bile and urine proteomic models

**Fig. 3:**
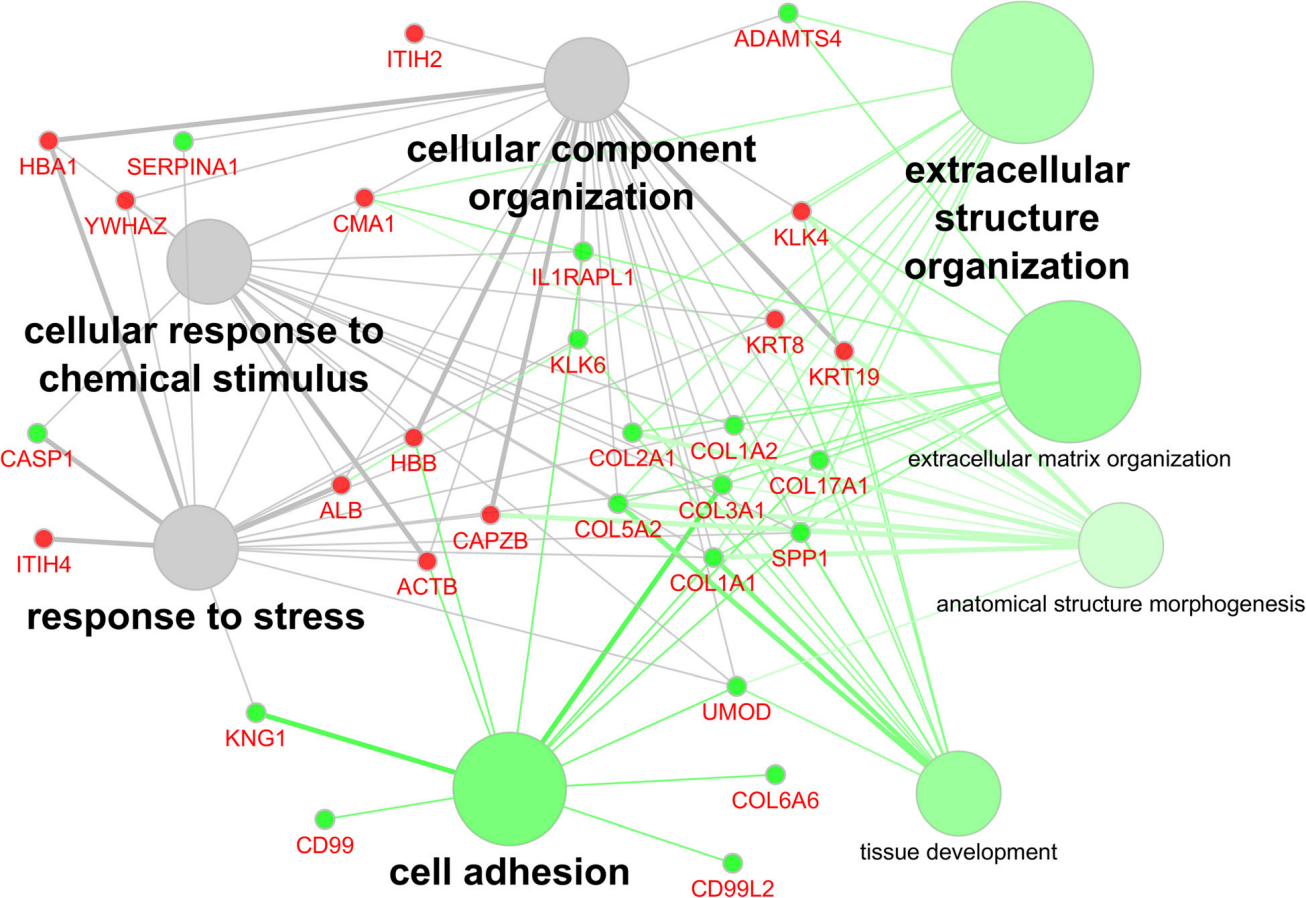
Functional association of proteins from which the cholangiocarcinoma (CCA) peptide markers included in the bile and urine proteomic models are derived together with the proteases predicted to be responsible for the generation of these peptides. Proteins were analyzed by functional Gene Ontology biological process group-clustering using CytoScape’s ClueGO plug-in (CytoScape v2.8.3, ClueGO v1.5). Enriched GO-terms are represented as circles, and lines denote the relationship between these terms as functional groups. Line thickness and font-size are directly correlated with the statistical significance of terms and relationships (all with *p* <  0.05 after Bonferroni-adjustment for multiple testing correction). Statistically significant molecules primarily identified and modulated in bile are marked by a small red circle, whereas urinary proteins are small green circles. The larger circles are associated with GO terms and the coloration is either urine (green) or modulated in both sources (grey)

Ontology analysis of associated biological terms using the GO term group “biological process” of statistically significant disease-associated biomarkers included in bile or urine panels, as well as predicted proteases, is shown in Fig. 3. Overlap between both sample types include stress response, response to chemical stimulus, and cellular component organization. Urine specific associated terms indicated a strong involvement in the extracellular matrix (ECM) and its organization, including cell adhesion, tissue development and anatomical structure morphogenesis. The latter two are indicative of a destructive event in the tissue, coupled with cognate proteases and modulatory incidents linked to the ECM, and a potential attempt of tissue repair. This in turn may impact on the organization of the ECM and the ability of cells to adhere appropriately, causing stress response and associated release of chemical compounds. Activity of the above proteases is directly implicated, as they are potential drivers of these events in the disease setting.

### Differential tissue gene expression profiling of precursor proteins and proteases associated with the bile and urine CCA peptide markers

Transcriptomic data from different gene arrays including CCA tumor tissue, paired non-malignant tissue and tissue from benign liver lesions, fibrous tissue, and normal bile ducts (all retrieved from the NCBI GEO database) were analyzed for differential expression of the protein precursors from which the bile and urine CCA peptide markers are derived as well as of the proteases predicted to be responsible for their specific cleavage. As presented in Additional file 1: Table S3, including the annotated results on Gene expression profiling data, for all parental proteins and the predicted proteases, tissue expression was confirmed at the transcript level. In terms of differential expression at the transcriptomics level, increased expression of YWHAZ, ACTB, F-actin-capping protein subunit β (CAPZB), the keratins 8 (KRT8) and 19 (KRT19), the collagen chains α-1(I), α-1(III) (COL3A1), α-2(I) (COL1A2), α-2(V) (COL5A2), osteopontin (SPP1) and KLK6, and decreased expression of HBA1, HBB, ITIH2, ITIH4, ALB, kininogen-1 (KNG1), collagen chain α-6(VI) (COL6A6), CD99 and CD99 antigen-like protein 2 (CD99L2), was identified in the CCA tumor tissue compared to paired non-tumor tissue and tissue from benign liver lesions or normal bile ducts.

Importantly, for cytoskeletal proteins, like ACTB or CAPZB, consistently higher expression was identified at the transcriptomics level, confirmed also by increased excreted peptides in bile from patients with CCA. This observation was also concordant with YWHAZ tissue expression at the transcriptomics level and its peptides excreted in bile. ITIH2 was consistently downregulated in transcriptomics and bile excretion proteomics data, whereas ITIH4 showed a decrease in the transcriptomics data sets and an increase in the peptide excretion profiles.

Consistent observations regarding several collagen fragments, such as COL1A2, COL3A1, COL5A2, collagen chain α-1(XVII) (COL17A1), were evident across the different transcriptomics comparisons and in urine proteomic analysis, all indicative of both increased transcript levels and increased peptide excretion in urine. Furthermore, consistent decrease at the transcriptomics level and in the urine peptide profiles was demonstrated for CD99, which is negatively regulating Cdc42 in tumour cells. The same consistent reduction was observed for CD99L2 and PGMRC1.

Nevertheless, some transcriptomics data were discordant to the regulation of the bile and urine peptides, as indicated in Additional file 1: Table S3. To investigate the disagreements in more detail, gene expression was investigated based on the “Pan-Cancer Analysis of Whole Genomes” project RNA-Seq mRNA database as a second source of information allowing a more in-depth analysis of the proteins differential expression profiles in both CCA and HCC tumors, but also in tumor-adjacent and normal liver tissue. As revealed by the box-and-whisker plot representation for each individual parental protein or protease shown in the Additional file 1: Figures S1 to S24, the disagreement between transcriptomics and proteomics data can be partially explained by differences in the expression of several of these proteins in tissue adjacent to CCA compared to tissue of normal livers. Another remarkable finding is, that the vast majority of proteins from which the bile and urine CCA peptide markers are derived, not only showed differences to normal liver, but also to HCC. Yet, in respect to the in silico predicted proteases, tissue transcriptomics data sets were inconclusive.

### Literature survey for functional characteristics of the protein precursors of bile and urine peptide markers

We performed a literature search to identify processes associated with changes in the bile proteome reflecting local changes and those that are involved in the systemic spreading of CCA leading to the observed CCA-specific changes in the urinary proteome. Involvement of the CCA peptide markers (and their corresponding parental proteins) in the pathophysiological context of CCA but also cancer progression in general is presented in Additional file 1: Table S4 for biomarkers detected in bile, and in Additional file 1: Table S5 for biomarkers detected in urine. For 10 out of 16 sequenced peptides included in the bile and 26 out of 30 sequenced peptides included in the urine proteomic model we identified a direct connection of their parental proteins to CCA. The numbers increased to 16 biliary and 29 urinary peptide markers, if the literature search was extended to an involvement in cancer pathology in general. Parental proteins of the bile peptide markers, for which the involvement in CCA was described in literature, are the cytoskeletal keratins KRT8 and KRT19, the inter-α trypsin inhibitor heavy chains H2 and H4, ACTB, ALB, YWHAZ and CAPZB. In urine, peptide markers are derived from the following proteins with well-described associations to CCA pathology in literature: KNG1, FXYD2, α-1-antitrypsin (SERPINA1), interleukin-1 receptor accessory protein-like 1 (IL1RAPL1), CD99 antigen, SPP1 and the collagen chains COL1A1, COL17A1, COL1A2, COL2A1, COL5A2 and COL6A6. Specifically in CCA these proteins are involved in EMT, cancer associated fibroblast (CAF)-mediated tumor growth, tumor invasion and metastasis, regulation of immune responses and maintenance of homeostasis.

## Discussion

CCA is a devastating cancer arising from malignant transformation of cholangiocytes in the biliary ducts. Prognosis remains poor as CCA is often detected in an unresectable stage [26]. The main predisposing factor for CCA in Western countries is PSC, a chronic cholestatic liver disease often requiring liver transplantation [27]. Differentiation between malignant and benign strictures is important to determine the appropriate treatment, but challenging even for specialists, since CCA and chronic or acute inflammation frequently result in similar cholangiographic findings.

For patients with PSC, orthotopic liver transplantation is the only therapy recommended, which can only be performed in the absence of CCA. Incidental CCA is the main reason for poor PSC patient survival after liver transplantation due to fast tumor progression in response to post-transplant immunosuppression [28].

Although the specificity of endoscopy with brush cytology is generally close to 100%, sensitivity ranges from 50 to 60% [8, 29]. The same is true for “classical” clinical and laboratory parameters. Elevated transaminases, cholestatic parameters and bilirubin may reflect hepatobiliary inflammation and do not play a role for the detection of biliary malignancy. In fact, bilirubin, which is often raised in CCA, is not specific, as it can be dramatically increased in benign strictures such as secondary sclerosing cholangitis [30] or biliary stones [31]. Moreover, the tumor marker CA 19–9, related to the Lewis blood group antigens, is absent in 5–10% of the population (leading to false negative test results) and is also frequently raised in patients with benign biliary obstruction and therefore not considered as a CCA marker applicable in clinical practice [6]. The differentiation of benign and malignant strictures solely by imaging studies is not feasible since frequently unclear bile duct changes such as stenosis, irregularity or thickening of the wall are detected instead of clear tumor masses in CCA patients [8].

We recently established diagnostic peptide marker models in bile and urine to detect both local and systemic signs of CCA progression [9–11]. These peptide marker models are based on CCA-specific molecular changes and are a complementary approach significantly improving CCA diagnosis when combined with a search for tumor masses by endoscopy or imaging techniques, histological screening for tumor cells and/or detection of single laboratory parameters for liver function.

Previous studies were focused on determining the diagnostic accuracy of the proteomic peptide marker patterns and their combination. In the present study, the main focus was set on evaluating the impact of a positive test result on patient survival and the investigation of the pathophysiological relevance of the bile and urine peptide markers. This was achieved by an in-depth literature search, as well as an in silico prediction of the proteases responsible for the cleavage of the peptide markers and identification of the relevant pathways by system biology methods and network analysis.

By in silico protease mapping the proteases ADAMTS4, CMA1 and KLK4 were predicted to be increased in their activity in CCA, whereas CASP1 and KLK6 were predicted to be decreased. ADAMTS4 was reported to promote tumor growth in colorectal cancer and is associated with macrophage infiltration [32]. CMA1 is released by activated mast cells to degrade the ECM. Infiltration of mast cells is described in intrahepatic and locally advanced CCA [33, 34], where these cells promote CCA tumor growth by histamine release [35]. Moreover, our data indicate a reduced activity of KLK6 and an increased activity of KLK4 in CCA. This is in perfect agreement with the aberrant expression pattern of kallikrein-related peptidases being implicated in EMT, as an EMT suppressor and an EMT promoter, respectively [36]. Silencing of KLK6 expression in cell cultures resulted in molecular features resembling EMT [37], whereas in the case of KLK4 high expression transduces EMT-like effects [38].

In line with the prediction of EMT-promoting KLK’s are reports that some of the peptide biomarker-deduced proteins are connected to a change in epithelial cell polarity and transition to a mesenchymal phenotype. This accounts for the decreased expression of the epithelial cell surface markers KRT19 and KRT8 and increased expression of the cytoskeletal components ACTB and CAPZB, as well as the EMT inducer YWHAZ [39].

Down-regulation of peptides derived from the CD99 and CD99L2 surface molecules in CCA indicates decreased cell surface expression, resulting in a loss of intercellular adhesion [40], as a step of depolarized cells after undergoing EMT shedding off from the epidermal layer. However, peptide fragments of these adhesion receptors were observed in urine and not in bile, indicating a systemic rather than a local feature of CCA.

Aside from the loss of epithelial cell polarity and EMT, mast cell activation in tumor areas was identified by an in silico predicted CMA1 activity on the bile peptide markers as CCA-associated event. Activated mast cells may indicate development of a systemic CCA response via release of heparin at the tumor site and activation of the contact induced coagulation cascade [41]. This is further supported by the identification of a KNG1-derived peptide fragment in urine of CCA patients. KNG1 is cleaved during coagulation into the proinflammatory vasodilator bradykinin. Thus, vasoactive substances may be released into the circulation by the action of mast cells in CCA with an impact on kidney homeostasis. Mast cell involvement in CCA is further supported by the finding that some of the urinary peptides included in the CCA-specific urinary peptide marker pattern were also differentially regulated in other diseases with well described mast cell pathology [42–44], like inflammatory bowel disease [45], rheumatoid arthritis [45], cardiovascular [46] and chronic kidney diseases [47].

Support for activation of release of anti-inflammatory cytokines via the IL-1 signaling pathway is provided by increased urinary levels of peptide fragments derived from the TOLL/IL-1 receptor family member IL1RAPL1, which specifically connects IL-38 released from apoptotic cells [48] to innate and adaptive immune cell inhibition [49] via its activation of the JNK signaling pathway [50]. This antagonistic effect of IL-38 thereby restricts anti-tumor immunity by inhibition of pro-inflammatory cytokine release of macrophages [51] and TH17 response of tumor-infiltrating γδ T cells [52]. In this context, it is noteworthy that SERPINA1 as another precursor protein for a urinary CCA peptide marker was reported to increase IL-1 receptor antagonist expression during tissue injury [53].

The main source of urinary peptide biomarkers for CCA are collagen chains. In literature, cancer-associated fibroblasts (CAFs) are associated with increased expression of collagens, excessive production of ECM and development of fibrosis in the tumor stroma. Since collagen enriched stroma at the tumor site is an inducer of EMT in epithelial cells [54], CAF-mediated fibrosis of tumor tissue turns into a self-propelling process. Excessive ECM production by CAFs might together with the release of ECM-degrading proteases, like CMA1 from mast cells, be responsible for increased occurrence of collagen fragments in the urine of CCA patients.

Decreased urinary levels of peptide fragments of homeostasis regulating and immunoregulatory proteins such as FXYD2 [55] and UMOD [56], whose expression is under control of the cell fate determining transcriptional factor HNF1B [57–59], is evident in CCA compared to benign biliary diseases. Both FXYD2 and UMOD are highly, and the latter almost exclusively, expressed in the kidney, indicating tight transcriptional regulation. Thus, decreased urinary levels of FXYD2 and UMOD derived peptides may indicate inhibition of HNF1B transcriptional activity by a mediator released at the tumor site into the circulation. HNF1B inhibition triggers EMT in human cancer cell lines [60, 61]. However, how this is transferred to the kidney is unclear.

Since the kidney is a selective filter of blood components, thereby maintaining homeostasis, the composition of urine peptides derived from proteolytic processing in tissues and body fluids, released into and then filtered from blood, is dynamic and thus can serve as a sensitive sample matrix even for diseases not directly connected to the kidney. This was demonstrated for various non-kidney diseases, i.e. those affecting the heart, colon or vasculature [45, 46, 62]. In a recent study involving over 2000 datasets from individual urine samples, multiple urine peptides could be demonstrated as being associated with the occurrence of solid tumors, potentially suggesting specific changes in urine peptides as a result of tumor invasion and cancer associated inflammation [63]. Compared to blood even subtle changes in the urine proteome can be detected due to its lower concentration range of protein and peptide compounds. This provides the advantage of non-invasive monitoring of patients at risk for the development of CCA independent of endoscopy. Urine proteome analysis therefore may play a key role in the surveillance of patients with PSC, because these young patients are at special risk for CCA and the diagnosis or exclusion of such malignant transformation at an optimum time point is very crucial to define their priority on the waiting list for liver transplantation.

CCA-specific changes in the proteolytic environment provides a rationale for the finding that several peptides derived from the highly abundant biliary proteins HBA1, HBB and KRT8 are increased while others are decreased during CCA progression. Based on the N- and C-terminal sequence motifs of the CCA peptide markers we used Proteasix to predict which proteases may be responsible for these CCA-associated changes in the proteolytic environment as part of the process leading to the CCA peptide markers. Alternatively, increased or decreased expression of parental proteins may be responsible for changes of the peptide abundance. By comparing peptide levels with publicly available tissue transcriptomics data sets, we identified increased expression of YWHAZ, ACTB, CAPZB, SPP1 and the collagen chains COL1A2, COL17A1, COL3A1 and COL5A2 in the CCA tumor both in the NCBI GEO and in the Whole Cancer Genome data sets and in agreement with our peptide profiles. The same accounts for decreased expression of ITIH2, SERPINA1, PGRMC1, CD99 and CD99L2. A common observation was that for structural and cytoskeletal proteins, like actin and actin regulators, consistently increased tissue transcriptomics expression and peptide excretion was detectable. The same was also demonstrated for several collagen chains such as COL1A2, COL17A1, COL3A1, COL5A2, likely reflecting changes in the ECM re-organization. Negative tumour regulators like CD99, and biomarkers whose decreased expression has been correlated with other tumours, like PGMRC1 were identified with consistently decreased expression and excretion levels, too [64–66].

For several proteins, like ALB and KNG1, the discordance between low gene expression levels in CCA tumor tissue and increased peptide levels may be explained in part by increased expression of these proteins in tissue adjacent to the tumor.

Transcriptomics data were inconclusive for the predicted proteases ADAMTS4, CMA1, CASP1, KLK4 and KLK6. However, regulation of these proteases typically occurs on the protein level, e.g. via prodomain removal in the case of ADAMTS4 [67], release from mast cell granules in the case of CMA1 [68] or self-cleavage in the case of CASP1 [69]. Regulation of proteases is complex and tightly controlled by different mechanisms on the protein level such as conversion from an inactive precursor, allosteric inhibition by small molecules, storage in granules or post-translational modifications [70]. Moreover, the proteases with decreased activity in CCA, namely CASP1 and KLK6, might be more prevalent in benign biliary disorders and in this respect the available transcriptomics data is rather sparse. In fact, CASP1 is a component of the inflammasome complex NRLP3 which is involved in fibrotic and sclerotic liver diseases such as liver fibrosis, PSC and biliary obstruction [71, 72].

When investigating the overlap between transcriptomics and proteomics profiling data, several studies found a significant, yet modest correlation reflected by Spearman rank values in the range of 0.45 to 0.75 [73–76]. Reasons for this consistent finding may be phenotypic effects of protein translation and post-translational modifications. For endogenous peptides, which emerge from degradation of proteins by certain proteases, an even higher level of complexity is introduced by the dynamic nature and plasticity of the proteolytic environment leading to increased occurrence of certain peptides from a particular protein precursor while other peptides from that protein may even disappear.

The fact that combination of the proteomic patterns from bile and urinary peptide profiles, are able to detect CCA with high accuracy (as proven in clinical studies), already indicates a relation of the included peptide biomarkers to both on-site and peripheral changes of CCA progression. Following up on this observation, integration of the peptide biomarkers by systems biology approaches and compilation to the literature provide further support for the involvement of CCA-related pathological processes, such as EMT. Gaining insights into a pathophysiological relevance of biomarkers is of high importance, especially when considering their implementation, as it is required for qualification of proteomics-based tests. Based on these findings, further investigations using molecular biology techniques might be performed to investigate in depth, the exact molecular mechanisms involved in CCA progression and might result in new molecular targets for therapeutic intervention.

### Limitations of the study

A limitation of the study is the fact that all patients were from one clinical center. However, this should have no impact on the results of our systems biology analyses and the significance of our biomarkers for CCA due to the large patient numbers and since they resemble a representative cross-sectional cohort of therapy-naïve patients covering all relevant disease etiologies and disease features.

A further limitation of the study may be that the majority of the data profiles were acquired during the previous investigations. As the established CE-MS analytical protocols (including standardized running conditions and calibration over internal standards) produce highly reproducible profiles, the data sets are fully comparable from the technical point of view. Therefore, interpretation of the previously acquired data sets supplemented with those from newly recruited patients in this follow-up investigation is not associated with technical variability.

## Conclusions

Bile and urine proteomic analysis enabled identification of specific peptides and the generation of classifiers that show a highly significant association, especially in combined bile and urine proteome analysis. This is further depicted to patient overall survival. By combining in silico protease prediction, network cluster analysis and a systematic literature survey, the CCA-specific bile and urinary peptides can be linked to the underlying pathophysiology. This resulted in the hypothesis that resident innate immune cells such as mast and stromal cells may be responsible for specific changes in the proteolytic environment associated with CCA progression. The mast cell-specific CMA1 and the stromal cell-derived KLK4, KLK6 and ADAMTS4 proteases were predicted to be associated with the peptides included in the bile and urine peptide marker pattern for CCA. The release of proteases, ECM components, inflammatory cytokines, proangiogenic and vasoactive factors into the circulation provides a way how local changes in the tumor microenvironment results in systemic manifestation of CCA progression, possibly even at early stages. The results provide a rationale for the observed excellent performance of urine proteome analysis for non-invasive CCA monitoring of risk patients or together with bile proteome analysis for a more accurate CCA diagnosis at the date of endoscopy.

## Supplementary information

**Additional file 1: Table S1.** Clinical and demographic data of patients analyzed in this study by combined bile and urine proteome analysis for CCA diagnosis. **Table S2.** Observed and in silico predicted protease associations to the N- and C-terminal amino acid sequence motifs of the bile and urine peptides included in the bile and urinary proteomic models for CCA diagnosis. **Table S3.** Transcriptomics data table summarizing the results from differential gene expression analysis for all parental proteins and predicted proteases associated with the bile and urine CCA peptide markers. **Figures S1-S24.** Box-and-whisker plot representations of RNA-seq mRNA levels given as “transcripts per million” (TPM) for all parental proteins and predicted proteases associated with the bile and urine CCA peptide markers in 18 CCA, 18 CCA-adjacent, 100 HCC, 53 HCC-adjacent and 35 normal liver tissue replicates. **Table S4.** Physiological and cancer-specific pathological implications of the parental proteins from which the bile peptide markers for CCA diagnosis by bile proteome analysis are derived. **Table S5.** Physiological and cancer-specific pathological implications of the parental proteins from which the urine peptide markers for CCA diagnosis by urine proteome analysis are derived.

## Data Availability

The datasets used and/or analysed during the current study are available from the corresponding author on reasonable request.

## Abbreviations

ACTB: Cytoplasmic actin 1
ADAMTS4: A disintegrin and metalloproteinase with thrombospondin motifs 4
AUC: Area under the curve
BBD: Benign biliary disease
BPA: Bile proteome analysis
CA 19–9: Carboanhydrate 19–9
CAF: Cancer-associated fibroblast
CAPZB: F-actin-capping protein subunit β
CASP1: Caspase-1
CCA: Cholangiocarcinoma
CD99: CD99 antigen
CD99L2: CD99 antigen-like protein 2
CE-MS: Capillary electrophoresis-mass spectrometry
CI: Confidence interval
CMA1: Chymase
COL17A1: Collagen chain α-1(XVII)
COL1A2: Collagen chain α-2(I)
COL3A1: Collagen chain α-1(III)
COL5A2: Collagen chain α-2(V)
COL6A6: Collagen chain α-6(VI)
ECM: Extracellular matrix
EMT: Epithelial-to-mesenchymal transition
FXYD2: Na^+^/K^+^ ATPase subunit γ
GO: Gene ontology
HBA1: Hemoglobin subunit α
HBB: Hemoglobin subunit β
IL1RAPL1: Interleukin-1 receptor accessory protein-like 1
ITIH2: Inter-α-trypsin inhibitor heavy chain H2
ITIH4: Inter-α-trypsin inhibitor heavy chain H4
KLK4: Kallikrein-4
KLK6: Kallikrein-6
KNG1: Kininogen-1
KRT19: Keratin 19
KRT8: Keratin 8
PGMRC1: Membrane-associated progesterone receptor component 1
PSC: Primary sclerosing cholangitis
ROC: Receiver operating characteristics
SERPINA1: α-1-antitrypsin
SPP1: Osteopontin
UMOD: Uromodulin
UPA: Urine proteome analysis
YWHAZ: 14–3-3 ζ/δ protein
