# Supplementary material for: Bile and urine peptide marker profiles: access keys to molecular pathways and biological processes in cholangiocarcinoma
Source: J Biomed Sci. 2020 Jan 3;27:13. doi: 10.1186/s12929-019-0599-5 (PMC6941325; doi:10.1186/s12929-019-0599-5)
Supplement: Supplementary file 1 — Additional file 1: Table S1. Clinical and demographic data of patients analyzed in this study by combined bile and urine proteome analysis for CCA diagnosis. Table S2. Observed and in silico predicted protease associations to the N- and C-terminal amino acid sequence motifs of the bile and urine peptides included in the bile and urinary proteomic models for CCA diagnosis. Table S3. Transcriptomics data table summarizing the results from differential gene expression analysis for all parental proteins and predicted proteases associated with the bile and urine CCA peptide markers. Figures S1-S24. Box-and-whisker plot representations of RNA-seq mRNA levels given as “transcripts per million” (TPM) for all parental proteins and predicted proteases associated with the bile and urine CCA peptide markers in 18 CCA, 18 CCA-adjacent, 100 HCC, 53 HCC-adjacent and 35 normal liver tissue replicates. Table S4. Physiological and cancer-specific pathological implications of the parental proteins from which the bile peptide markers for CCA diagnosis by bile proteome analysis are derived. Table S5. Physiological and cancer-specific pathological implications of the parental proteins from which the urine peptide markers for CCA diagnosis by urine proteome analysis are derived. [file 12929_2019_599_MOESM1_ESM.pdf]

Table S1. Clinical and demographic data of patients analyzed in this study by combined bile and urine proteome analysis for CCA diagnosis.

**Patient group**	**Malignant biliary stricture case groups**		**Benign biliary stricture control groups**			
	**CCA**	**CCA on top of PSC**	**PSC**	**BBD**		
Patients, n	33	19	57	19		
**Type of biliary disease (n)**						
Choledocholithiasis				10		
Chronic pancreatitis				3		
SSC				3		
CBD dilatation				3		
**Clinical and demographic parameters (normal values)**					**P for malignant to benign group differences***	**AUC for CCA diagnosis**
Age, years, mean/range	63/35-85	46/22-68	43/21-83	60/35-84	0.0004	0.68
Female/male, n/n	11/22	4/15	13/44	8/11	1.00	n.d.
Alkaline phosphatase (35 – 104 U/L), mean/range	395/61-1023	342/128-670	300/47-1475	251/51-1173	0.001	0.68
γ-Glutamyltransferase (< 39 U/L), mean/range	521/10-2212	379/83-937	352/15-3638	304/30-1340	0.002	0.67
Bilirubin (< 17 µmol/L), mean/range	131/4-502	112/5-506	48/4-260	30/5-126	0.006	0.65
Leucocyte count (4-10/nL), mean/range	8.5/3.6-20.8	8.5/0.8-18.0	7.3/1.0-20.0	6.4/2.3-10.9	0.15	0.58
C-reactive protein (< 8 mg/L), mean/range	45/1-184	33/3-215	14/1-91	28/1-190	0.002	0.67
Alanine aminotransferase (< 35 U/L), mean/range	96/14-551	205/27-716	90/15-548	56/16-205	0.031	0.62
Aspartate aminotransferase (< 32 U/L), mean/range	90/18-333	169/23-767	77/6-378	52/24-110	0.004	0.66
Carbohydrate antigen 19-9 (< 27 kU/L), mean/range	3562/1-81243	785/1-3955	56/1-293	146/1-543	<0.0001	0.75
**Number of detected peptides**						
in urine, mean/range	1901/750-3462	2113/1033-3800	2036/946-3036	1859/1270-2940	0.69	0.52
in bile, mean/range	1562/320-4158	1827/423-4488	1653/327-4165	2018/491-3458	0.42	0.54
**Classification factors by proteome analysis**						
Bile proteome analysis (BPA)	0.59/-1.24-2.28	0.14/-1.74-1.65	-0.36/-1.49-1.16	0.05/-1.12-1.40	<0.0001	0.79
Urine proteome analysis (UPA)	-0.11/-2.41-2.74	0.22/-0.82-3.19	-1.49/-3.13-1.09	-1.48/-2.64-0.27	<0.0001	0.90
Combined BPA and UPA	2.40/-3.14-10.29	2.44/-3.73-13.05	-2.98/-9.16-4.69	-2.38/-6.08-0.94	<0.0001	0.92

*Two-tailed probability for continuous data and significance level by Fisher exact test for categorical data.
Abbreviations: AUC, area under the curve; BBD, benign biliary disease; CBD, common bile duct; CCA, cholangiocarcinoma; n.d., not determined; PSC, primary sclerosing cholangitis; SSC, secondary sclerosing cholangitis.

Table S2. Observed and *in silico* predicted protease associations to the N- and C-terminal amino acid sequence motifs of the bile and urine peptides included in the bile and urinary proteomic models for CCA diagnosis. Confirmed cleavage site associations are based on the MEROPS peptide database, whereas *in silico* protease prediction was done by the software tool Proteasix.

Evidence level*	Biofluid	CCA peptide marker sequence	Parental protein	Protease cleavage at the peptide's N-terminal end†	Peptide sequence including flanking cleavage motifs	Protease cleavage at the peptide's C-terminal end†
Observed	Bile	173-FYAPELLFFAK-183	Serum albumin	MEP1B (M12.004)[1]	RHPY.**FYAPELLFFAK**	
		2-VHLTPEEKSAVTA-14	Hemoglobin subunit β	unknown peptidase[2]	M.**VHLTPEEKSAVTA**.LWGK	CAPN1 (C02.001)[3] /CTSD (A01.009)[4]
		28-SVTEQGAELSNEER-41	14-3-3 protein ζ/δ	CTSL (C01.032)[5] / CTSK (C01.036)[5] / CTSS (C01.034)[5] / CTSV (C01.009)[5] / PRSS1 (S01.151)[6]	ACMK.**SVTEQGAELSNEER**.NLLS	PRSS1 (S01.151)[6]
	Urine	26-DFDDFNLED-34	CD99 antigen-like protein 2	unknown peptidase[2]	RGSG.**DFDDFNLED**	
		97-DGVSGGEGKGGSDGGGSHRKEGEEADAPGVIPGIVG-132	CD99 antigen	MEP1B (M12.004)[7]	ADLA.**DGVSGGEGKGGSDGGGSHRKEGEEADAPGVIPGIVG**	
Predicted	Bile	27-AEALERmFL-35	Hemoglobin subunit α	ADAMTS4 (M12.221)	GEYG.**AEALERmFL**.SFPT	CMA1 (S01.140) / KLK4 (S01.251)
		173-FYAPELLFFAK-183	Serum albumin	CMA1 (S01.140)	RHPY.**FYAPELLFFAK**	
		179-DLAGRDLTDYLm-190	Actin, cytoplasmic 1	CMA1 (S01.140) / ADAMTS4 (M12.221) / KLK4 (S01.251)	ILRL.**DLAGRDLTDYLm**	
		28-SVTEQGAELSNEER-41	14-3-3 protein ζ/δ		**SVTEQGAELSNEER**.NLLS	ADAMTS4 (M12.221)
		381-ILLTDGDPTVGETNPR-396	Inter-α-trypsin inhibitor heavy chain H4		**ILLTDGDPTVGETNPR**.SIQN	KLK4 (S01.251)
		43-FESFGDLSTPDAVmGNPK-60	Hemoglobin subunit β	CMA1 (S01.140)	TQRF.**FESFGDLSTPDAVmGNPK**	
	Urine	589-SGSVIDQSR-597	Uromodulin	ADAMTS4 (M12.221) / KLK6 (S01.236)	TRFR.**SGSVIDQSR**.VLNL	CASP1 (C14.001) / KLK6 (S01.236)
		26-DFDDFNLED-34	CD99 antigen-like protein 2		**DFDDFNLED**.AVKE	ADAMTS4 (M12.221) / CASP1 (C14.001)
		613-DGEAGAQGPpGPA-625	Collagen α-1(I) chain	ADAMTS4 (M12.221) / KLK6 (S01.236)	PAGK.**DGEAGAQGPpGPA**	
		657-KpGEQGVpGDLG-668	Collagen α-1(I) chain	ADAMTS4 (M12.221)	GEAG.**KpGEQGVpGDLG**	
		1007-GPpGESGREGApG-1019	Collagen α-1(I) chain		**GPpGESGREGApG**.AEGS	ADAMTS4 (M12.221)
		1701-GLpGEmGSpGEPG-1713	Collagen α-6(VI) chain	CASP1 (C14.001)	MISA.**GLpGEmGSpGEPG**	
		918-RpGEVGPpGPpGP-930	Collagen α-1(I) chain	ADAMTS4 (M12.221)	GPAG.**RpGEVGPpGPpGP**.AGEK	ADAMTS4 (M12.221)
		651-pPGEAGKpGEQGVP-664	Collagen α-1(I) chain		**pPGEAGKpGEQGVP**.GDLG	
		543-SpGSPGPDGKTGPpGPAG-560	Collagen α-1(I) chain	ADAMTS4 (M12.221) / CASP1 (C14.001)	GLTG.**SpGSPGPDGKTGPpGPAG.**QDGR	ADAMTS4 (M12.221)
		54-SGDSDDDEPPPLPRL-68	Membrane-associated progesterone receptor component 1		**SGDSDDDEPPPLPRL**.KRRD	ADAMTS4 (M12.221)
		288-EpGSpGENGApGQMGPR-304	Collagen α-1(I) chain	ADAMTS4 (M12.221)	GPKG.**EpGSpGENGApGQMGPR**.GLPG	CASP1 (C14.001)
		1084-ApGPQGPRGDkGETGERG-1101	Collagen α-1(III) chain		**ApGPQGPRGDkGETGERG**.AAGI	ADAMTS4 (M12.221)
		550-DGKTGpPGPAGQDGRPGPpGppG-572	Collagen α-1(I) chain	ADAMTS4 (M12.221)	SPGP.**DGKTGpPGPAGQDGRPGPpGppG.**ARGQ	ADAMTS4 (M12.221)
		189-kGQpGApGVkGEpGApGENGTpGQTGARG-217	Collagen α-2(I) chain		**kGQpGApGVkGEpGApGENGTpGQTGARG**.LPGE	ADAMTS4 (M12.221)
		780-DKGESGpSGpAGpTGARGApGDRGEpGppG-809	Collagen α-1(I) chain	ADAMTS4 (M12.221)	GAPG.**DKGESGpSGpAGpTGARGApGDRGEpGppG**	
		188-LkGQpGApGVKGEpGApGENGTPGQTGARG-217	Collagen α-2(I) chain	ADAMTS4 (M12.221)	GLDG.**LkGQpGApGVKGEpGApGENGTPGQTGARG.**LPGE	ADAMTS4 (M12.221)
		188-LkGQpGApGVkGEpGApGENGTpGQTGARG-217	Collagen α-2(I) chain	ADAMTS4 (M12.221)	GLDG.**LkGQpGApGVkGEpGApGENGTpGQTGARG.**LPGE	ADAMTS4 (M12.221)
		194-ESEELNGAYKAIPVAQDLNAPSDWDSRGKDSYETSQL-230	Osteopontin		**ESEELNGAYKAIPVAQDLNAPSDWDSRGKDSYETSQL**.DDQS	ADAMTS4 (M12.221)

* The MEROPS peptidase database available at https://www.ebi.ac.uk/merops/index.shtml was screened for experimentally confirmed protease to cleavage site associations for the differentially expressed peptides leading to the list of observed assignments in the upper part of the table . The lower part of the table consist of *in silico* predicted protease assignments made by the online software tool Proteasix which is based on the specificity weight matrices provided by the MEROPS peptidase database . The MEROPS specificity matrix is a measure for the frequency of specific amino acids at each position in the experimentally confirmed cleavage site of a given protease. Proteasix transforms the specificity matrices to probability matrices by dividing the number of occurrences for each of the 20 possible amino acids with the total number of experimental observations. From the 25 billion combinations only those were kept with a propability score greater than the 99th percentile. Moreover, in order to eliminate false positive predictions, a high confidence threshold was calculated based on six thousand random sequences generated by the Expassy RandSeq tool and only those protease to cleavage site associations were considered below this threshold.

† Proteases were restricted to those of human origin. Proteases are specified with their gene names together with their MEROPS classification ID's in parentheses.

Reference list: [1] Bertenshaw GP, Turk BE, Hubbard SJ, Matters GL, Bylander JE, Crisman JM, et al. Marked differences between metalloproteases meprin A and B in substrate and peptide bond specificity. J Biol Chem. 2001;276:13248-55. **[2]** López-Pelegrín M, Cerdà-Costa N, Cintas-Pedrola A, Herranz-Trillo F, Bernadó P, Peinado JR, et al. Multiple stable conformations account for reversible concentration-dependent oligomerization and autoinhibition of a metamorphic metallopeptidase. Angew Chem Int Ed Engl. 2014;53:10624-30. **[3]** Shinkai-Ouchi F, Koyama S, Ono Y, Hata S, Ojima K, Shindo M, et al. Predictions of Cleavability of Calpain Proteolysis by Quantitative Structure-Activity Relationship Analysis Using Newly Determined Cleavage Sites and Catalytic Efficiencies of an Oligopeptide Array. Mol Cell Proteomics. 2016;15:1262-80. **[4]** Brindley PJ, Kalinna BH, Wong JY, Bogitsh BJ, King LT, Smyth DJ, et al. Proteolysis of human hemoglobin by schistosome cathepsin D. Mol Biochem Parasitol. 2001;112:103-12. **[5]** Vidmar R, Vizovišek M, Turk D, Turk B, Fonović M. Protease cleavage site fingerprinting by label-free in-gel degradomics reveals pH-dependent specificity switch of legumain. EMBO J. 2017;36:2455-65. **[6]** Schilling O, Overall CM. Proteome-derived, database-searchable peptide libraries for identifying protease cleavage sites. Nat Biotechnol. 2008;26:685-94. **[7]** Becker-Pauly C, Barré O, Schilling O, Auf dem Keller U, Ohler A, Broder C, et al. Proteomic analyses reveal an acidic prime side specificity for the astacin metalloprotease family reflected by physiological substrates. Mol Cell Proteomics. 2011;10:M111.009233.

Table S3. Transcriptomics data table summarizing the results from differential gene expression analysis for all parental proteins and predicted proteases associated with the bile and urine CCA peptide markers. All tissue transcriptomics data sets were retrieved from the NCBI Gene Expression Omnibus (GEO) database (https://www.ncbi.nlm.nih.gov/geo/) and analyzed with GEO2R (https://www.ncbi.nlm.nih.gov/geo/geo2r/). The analysis was based on the following gene arrays: GSE76297 for CCA tumour and paired non-malignant tissue, GSE26566 for CCA tumour, matched non-malignant surrounding liver and normal bile duct tissue, GSE32879 for intrahepatic CCA tumour, matched non-malignant surrounding liver and benign liver lesions tissue derived from focal nodular hyperplasia patients, and GSE45001 for laser capture microdissected fibrous tissue from intrahepatic CCA tumour and non-malignant tissue. Differential tissue expression is reported as fold-change in CCA tissue versus control, supplemented by the adjusted p-value for false discovery rate and the identification number per gene array. Fold change ratios >1.00, representing increased expression in CCA tumour tissue, are highlighted in red font, whereas those <1.00, representing decreased expression in CCA tumour tissue, are highlighted in blue font. False discovery rate-adjusted p-values <0.05 are marked in black, those >0.05 in grey font.

Protein specification			Differential tissue transcriptomics analysis reported as fold change / adj. p-value / ID						Representation at the proteomic level	
			GSE76297	GSE26566		GSE32879		GSE45001		
Protein name	UniProt ID	Gene	CCA tumor tissue (n=91) - vs - Paired non-tumor tissue (n=92)	CCA tumor tissue (n=104) - vs - Matched non-cancerous surrounding liver tissue (n=59)	CCA tumor tissue (n=104) - vs - Unmatched normal intrahepatic bile duct (n=6)	Intrahepatic CCA (n=16) - vs - Paired non-cancerous liver tissue (n=7)	Intrahepatic CCA (n=16) - vs - Unmatched benign liver lesions (FNH, n=5)	CCA tumoral stroma (n=10) - vs - Matched non-tumoral stroma (n=10)	Role	Differential peptide expression in CCA vs. PSC/BBD
14-3-3 protein ζ/δ	P63104	YWHAZ	2.37 / 2.65E-63 / TC08001485.hg.1	2.71 / 2.08E-27 / ILMN_1801928<br>3.23 / 4.03E-07 / ILMN_1669286	2.23 / 1.47E-03 / ILMN_1801928<br>3.53 / 5.67E-02 / ILMN_1669286	3.13 / 8.56E-09 / 8152096	2.53 / 5.64E-06 / 8152096	1.96 / 4.49E-02 / A_32_P198923<br>2.00 / 1.03E-01 / A_32_P226149	Source of bile peptide marker(s)	Up
Actin, cytoplasmic 1	P60709	ACTB	1.59 / 1.88E-43 / TC07001120.hg.1	1.11 / 1.12E-04 / ILMN_1343294<br>1.83 / 9.13E-22 / ILMN_2038777<br>2.38 / 1.39E-17 / ILMN_1777296	1.04 / 7.93E-01 / ILMN_1343294<br>2.10 / 7.23E-05 / ILMN_2038777<br>1.83 / 6.01E-02 / ILMN_1777296	1.65 / 3.47E-03 / 8137979	1.49 / 5.90E-02 / 8137979	1.29 / 5.71E-01 / A_32_P137939<br>1.04 / 9.56E-01 / A_23_P135769		Up
F-actin-capping protein subunit β	P47756	CAPZB	1.35 / 2.28E-25 / TC01002300.hg.1	1.17 / 1.12E-03 / ILMN_1798980	1.22 / 2.95E-01 / ILMN_1798980	1.34 / 1.17E-01 / 7913169	1.23 / 4.90E-01 / 7913169	1.32 / 4.96E-01 / A_33_P3235568<br>1.23 / 6.67E-01 / A_23_P126752		Up
Hemoglobin subunit α	P69905	HBA1	0.51 / 6.34E-21 / TC16000008.hg.1	0.34 / 4.71E-07 / ILMN_1667796	0.88 / 9.32E-01 / ILMN_1667796	0.3 / 2.20E-04 / 7991766	0.87 / 7.77E-01 / 7991766	N/A		Down / Up
Hemoglobin subunit β	P68871	HBB	0.62 / 8.16E-17 / TC11001330.hg.1	0.35 / 2.78E-06 / ILMN_1769753	0.57 / 6.33E-01 / ILMN_1769753	0.23 / 4.86E-07 / 7946033	0.71 / 3.06E-01 / 7946033	0.04 / 1.94E-03 / A_23_P203558		Down / Up
Inter-α-trypsin inhibitor heavy chain H2	P19823	ITIH2	0.04 / 4.55E-48 / TC10000066.hg.1	0.11 / 2.39E-13 / ILMN_1808494	0.26 / 2.80E-01 / ILMN_1808494	0.11 / 2.82E-03 / 7926061	0.1 / 1.87E-02 / 7926061	0.05 / 2.96E-04 / A_23_P202053		Down
Inter-α-trypsin inhibitor heavy chain H4	Q14624	ITIH4	0.07 / 1.52E-57 / TC03003413.hg.1	0.09 / 1.98E-25 / ILMN_1718525	0.18 / 2.65E-02 / ILMN_1718525	0.04 / 1.10E-09 / 8088020	0.04 / 1.41E-07 / 8088020	0.02 / 2.41E-07 / A_23_P109881		Up
Keratin, type I cytoskeletal 19	P08727	KRT19	9.67 / 4.92E-57 / TC17002906.hg.1	25.28 / 1.02E-29 / ILMN_1753924	8.46 / 6.82E-03 / ILMN_1753924	6.71 / 3.73E-03 / 8015349	7.5 / 1.84E-02 / 8015349	2.58 / 2.57E-01 / A_23_P66798		Down
Keratin, type II cytoskeletal 8	P05787	KRT8	1.81 / 3.55E-23 / TC12001539.hg.1<br>1.61 / 2.46E-16 / TC08000415.hg.1	1.97 / 3.22E-09 / ILMN_1753584	3.27 / 2.17E-03 / ILMN_1753584	2.08 / 4.20E-03 / 7963567	1.71 / 8.57E-02 / 7963567	2.55 / 3.54E-01 / A_24_P67395<br>1.00 / 9.98E-01 / A_33_P3372368<br>1.00 / 9.98E-01 / A_23_P14072		Down / Up
Serum albumin	P02768	ALB	0.08 / 7.17E-30 / TC04000403.hg.1	0.27 / 8.38E-06 / ILMN_1682763	0.24 / 2.88E-01 / ILMN_1682763	0.12 / 8.32E-03 / 8095628	0.12 / 4.52E-02 / 8095628	0.01 / 1.02E-07 / A_23_P257834		Up
Uromodulin	P07911	UMOD	0.91 / 2.84E-07 / TC16000924.hg.1	1.12 / 6.26E-01 / ILMN_1740585	1.04 / 9.76E-01 / ILMN_1740585	0.92 / 3.78E-01 / 7999936	0.98 / 8.94E-01 / 7999936	0.84 / 8.11E-01 / A_23_P158775	Source of urine peptide marker(s)	Down
Kininogen-1	P01042	KNG1	0.03 / 1.54E-51 / TC03001024.hg.1	0.04 / 3.04E-17 / ILMN_1753729	0.06 / 4.82E-02 / ILMN_1753729	0.02 / 1.13E-05 / 8084679	0.02 / 3.52E-04 / 8084679	0.003 / 9.23E-07 / A_23_P212258		Up
CD99 antigen-like protein 2	Q8TCZ2	CD99L2	0.65 / 1.15E-36 / TC0X001486.hg.1	0.59 / 7.35E-10 / ILMN_1700681	0.55 / 5.95E-02 / ILMN_1700681	0.68 / 2.90E-02 / 8175647	0.82 / 4.56E-01 / 8175647	0.75 / 7.19E-01 / A_23_P253052		Down
Collagen α-1(I) chain	P02452	COL1A1	4.66 / 2.91E-45 / TC17001682.hg.1	14.62 / 2.65E-30 / ILMN_1701308	5.17 / 1.01E-02 / ILMN_1701308	8.5 / 1.36E-08 / 8016646	3.61 / 8.47E-06 / 8016646	22.47 / 7.37E-04 / A_33_P3304668		Down / Up
Collagen α-6(VI) chain	A6NMZ7	COL6A6	0.76 / 6.97E-32 / TC03000705.hg.1	N/A	N/A	0.56 / 4.15E-05 / 8082597	0.71 / 3.04E-03 / 8082597	N/A		Up
Na^+/K^+-ATPase γ subunit	P54710	FXYD2	0.77 / 8.85E-25 / TC11003508.hg.1	1.42 / 1.80E-01 / ILMN_1781045<br>1.89 / 1.06E-01 / ILMN_1715301	0.60 / 7.22E-01 / ILMN_1781045<br>0.27 / 3.32E-01 / ILMN_1715301	2.26 / 9.14E-02 / 7951966	0.80 / 4.15E-01 / 7951977	0.47 / 4.22E-01 / A_23_P161769<br>0.72 / 7.31E-01 / A_33_P3228445		Down
Membrane-associated progesteronereceptor component 1	O00264	PGRMC1	0.42 / 9.08E-60 / TC0X000580.hg.1	0.47 / 1.99E-16 / ILMN_1684771	0.72 / 4.01E-01 / ILMN_1684771	0.28 / 1.97E-04 / 8169617	0.32 / 5.84E-03 / 8169617	0.49 / 1.72E-01 / A_33_P3332130		Down
Collagen α-1(III) chain	P02461	COL3A1	3.91 / 1.26E-34 / TC02001105.hg.1	4.79 / 7.60E-20 / ILMN_1773079	2.95 / 5.48E-02 / ILMN_1773079	4.47 / 5.27E-06 / 8046922	2.69 / 5.31E-03 / 8046922	3.86 / 3.31E-01 / A_24_P935491		Up
Collagen α-2(I) chain	P08123	COL1A2	5.96 / 9.87E-49 / TC07000559.hg.1	8.06 / 3.13E-22 / ILMN_1785272	1.78 / 5.08E-01 / ILMN_1785272	7.75 / 1.02E-08 / 8134263	3.07 / 5.47E-05 / 8134263	6.59 / 2.94E-02 / A_24_P277934		Up
Collagen α-1(II) chain	P02458	COL2A1	1.02 / 4.29E-01 / TC12001440.hg.1	1.40 / 6.82E-01 / ILMN_1686538<br>0.65 / 7.43E-03 / ILMN_1742840	1.15 / 8.76E-01 / ILMN_1742840	1.06 / 6.32E-01 / 7962703	1.09 / 6.24E-01 / 7962703	N/A		Down
α-1-antitrypsin	P01009	SERPINA1	0.36 / 6.71E-36 / TC14001466.hg.1	0.73 / 4.24E-03 / ILMN_1745961<br>2.60 / 6.70E-06 / ILMN_1764980<br>0.68 / 1.55E-01 / ILMN_1686098	0.67 / 4.72E-01 / ILMN_1745961<br>2.43 / 2.98E-01 / ILMN_1764980<br>0.73 / 8.33E-01 / ILMN_1686098	0.51 / 1.73E-02 / 7981068	0.48 / 5.18E-02 / 7981068	0.17 / 1.36E-03 / A_23_P218111<br>0.19 / 2.27E-02 / A_33_P3289659		Down
Collagen α-1(XVII) chain	Q9UMD9	COL17A1	N/A	14.32 / 1.83E-06 / ILMN_1799105<br>13.64 / 4.04E-11 / ILMN_1651282	9.99 / 3.52E-01 / ILMN_1799105<br>3.43 / 4.65E-01 / ILMN_1651282	1.24 / 6.17E-01 / 7936144	1.4 / 6.17E-01 / 7936144	N/A		Up
Collagen α-2(V) chain	P05997	COL5A2	2.46 / 2.39E-33 / TC02002612.hg.1	5.13 / 4.75E-21 / ILMN_1729117	2.43 / 1.53E-01 / ILMN_1729117	3.54 / 9.36E-05 / 8057620	2.59 / 1.43E-02 / 8057620	5.21 / 1.63E-01 / A_23_P33196		Up
Interleukin-1 receptor accessoryprotein-like 1	Q9NZN1	IL1RAPL1	0.94 / 8.15E-04 / TC0X000133.hg.1	0.66 / 2.82E-04 / ILMN_1756598	0.78 / 6.18E-01 / ILMN_1756598	1.09 / 6.56E-01 / 8166593	1.03 / 9.39E-01 / 8166593	N/A		Up
CD99 antigen	P14209	CD99	0.93 / 1.20E-02 / TC0X000014.hg.1<br>0.93 / 2.16E-02 / TC0Y000014.hg.1	0.93 / 4.06E-01 / ILMN_1785188	0.59 / 1.00E-01 / ILMN_1785188	0.83 / 2.39E-01 / 8176360	0.84 / 4.79E-01 / 8176360	0.77 / 6.57E-01 / A_24_P70993		Down
Osteopontin	P10451	SPP1	8.66 / 1.63E-33 / TC04000478.hg.1	8.63 / 9.98E-20 / ILMN_1651354	2.27 / 3.56E-01 / ILMN_1651354	53.74 / 9.66E-12 / 8096301	25.92 / 1.35E-09 / 8096301	10.06 / 1.34E-03 / A_23_P7313		Up
Kallikrein-4	Q9Y5K2	KLK4	0.88 / 5.98E-07 / TC19001761.hg.1	2.31 / 4.47E-01 / ILMN_1792363	N/A	0.89 / 2.65E-01 / 8038655	0.97 / 8.59E-01 / 8038655	N/A	Predicted protease	Up
Chymase	P23946	CMA1	0.83 / 1.74E-23 / TC14000979.hg.1	0.97 / 9.46E-01 / ILMN_1770772	2.25 / 5.14E-01 / ILMN_1770772	0.87 / 7.71E-02 / 7978343	0.78 / 2.84E-02 / 7978343	0.54 / 4.97E-01 / A_33_P3259708		Up
A disintegrin and metalloproteinase with thrombospondin motifs 4	O75173	ADAMTS4	1.06 / 3.39E-01 / TC01003421.hg.1	0.85 / 4.72E-01 / ILMN_1751489	0.13 / 4.26E-04 / ILMN_1751489	1.32 / 4.33E-01 / 7921821	1.58 / 2.80E-01 / 7921821	0.56 / 7.89E-01 / A_23_P360754		Up
Caspase-1 (Interleukin-1 converting enzyme)	P29466	CASP1	1.21 / 4.09E-04 / TC11003505.hg.1	0.93 / 5.72E-01 / ILMN_1742270<br>1.27 / 6.42E-01 / ILMN_1727762	0.68 / 4.69E-01 / ILMN_1742270<br>2.87 / 4.73E-01 / ILMN_1727762	1.19 / 5.21E-01 / 7951397	0.91 / 8.65E-01 / 7951397	1.13 / 8.91E-01 / A_23_P202978		Down
Kallikrein-6	Q92876	KLK6	1.96 / 4.71E-09 / TC19001764.hg.1	2.07 / 5.69E-05 / ILMN_1777541<br>26.72 / 1.10E-04 / ILMN_1780255	1.43 / 7.15E-01 / ILMN_1777541	1.19 / 7.21E-01 / 8038683	1.29 / 7.59E-01 / 8038683	N/A		Down

Figures S1-S24. Box-and-whisker plot representations of RNA-seq mRNA levels given as "transcripts per million" (TPM) for all parental proteins and predicted proteases associated with the bile and urine CCA peptide markers in 18 CCA, 18 CCA-adjacent, 100 HCC, 53 HCC-adjacent and 35 normal liver tissue replicates. Transcriptomics data sets were retrieved from the "Pan-Cancer Analysis of Whole Genomes" project RNA-Seq mRNA repository available at https://www.ebi.ac.uk/. No RNA-seq data was available for UMOD, COL6A6, COL2A1, IL1RAPL1, KLK4, CMA1 and KLK6.

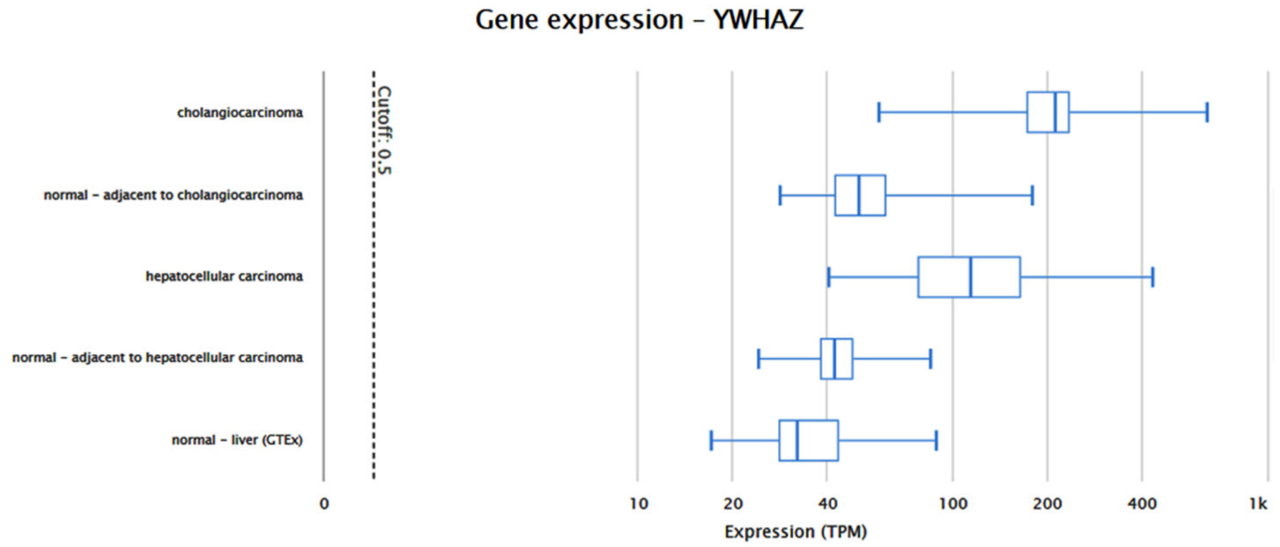

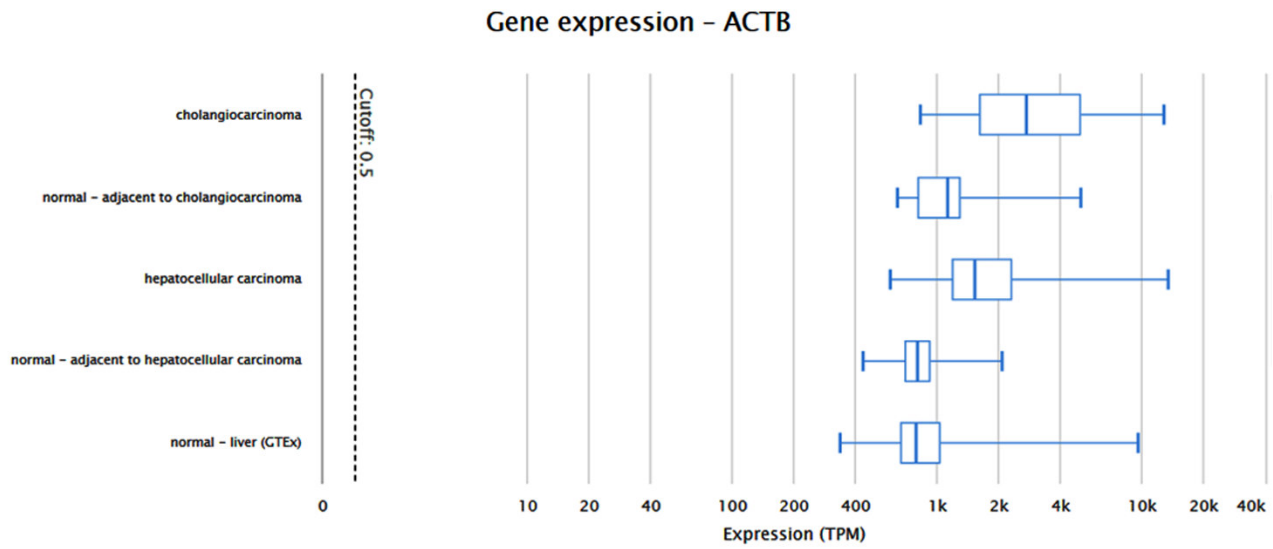

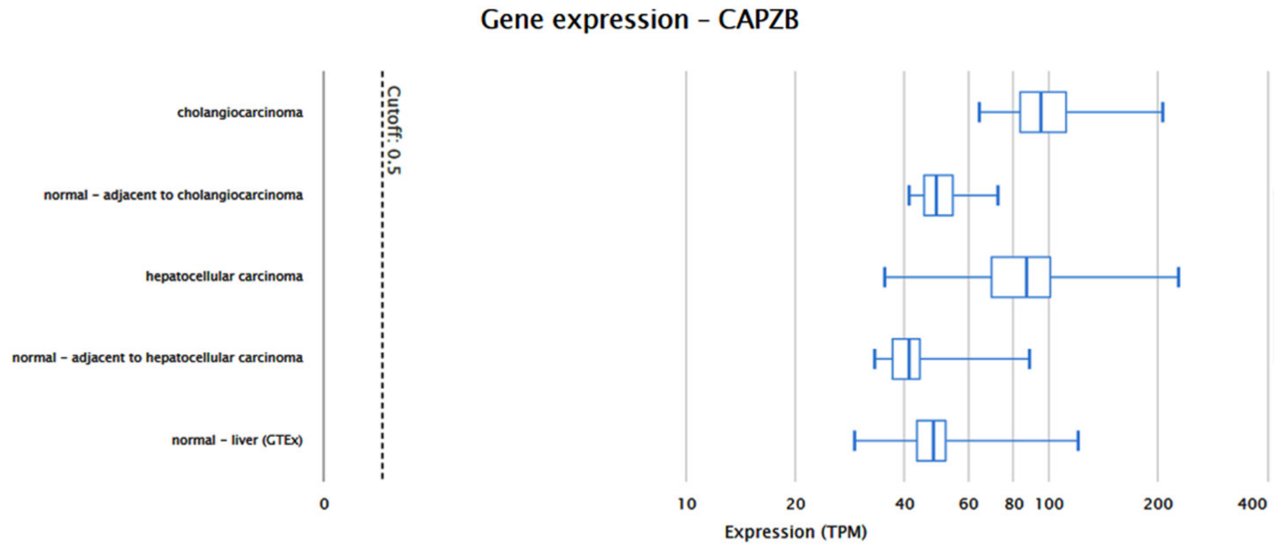

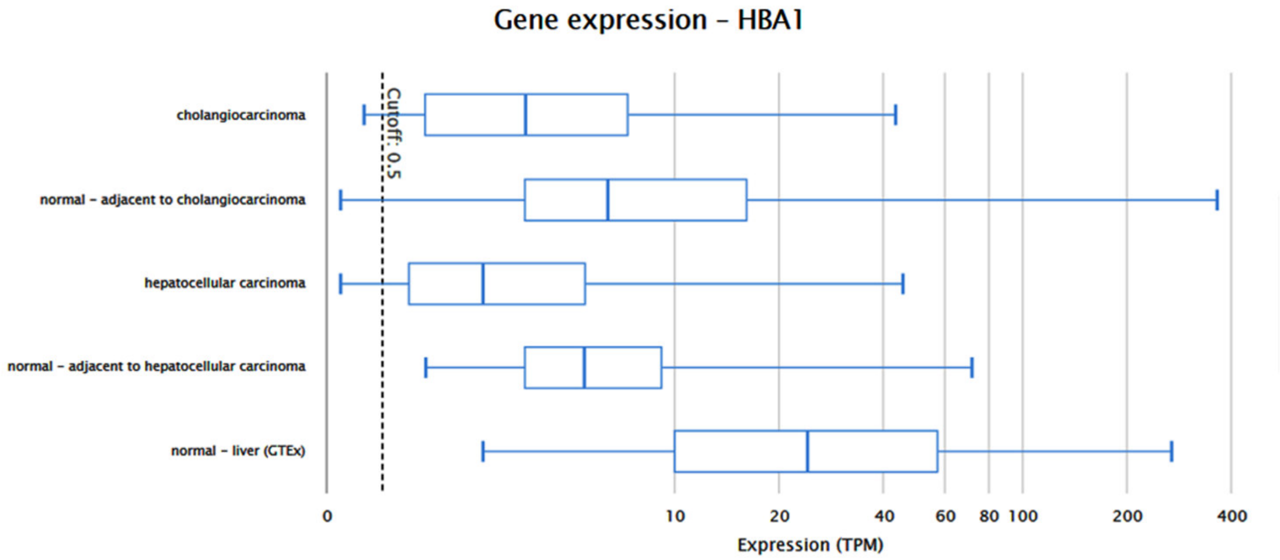

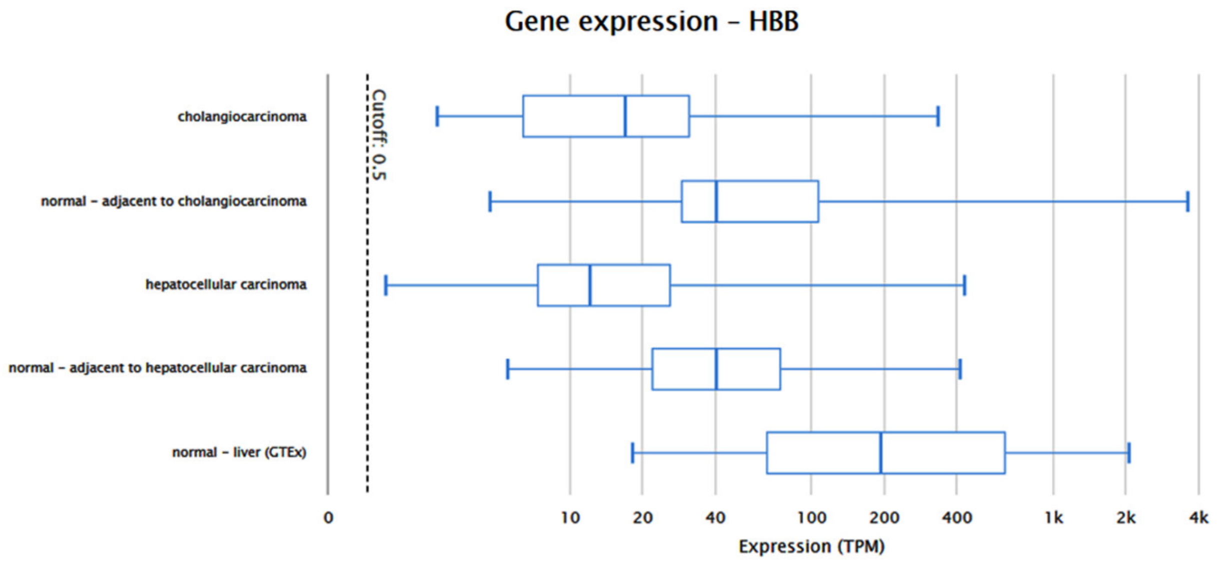

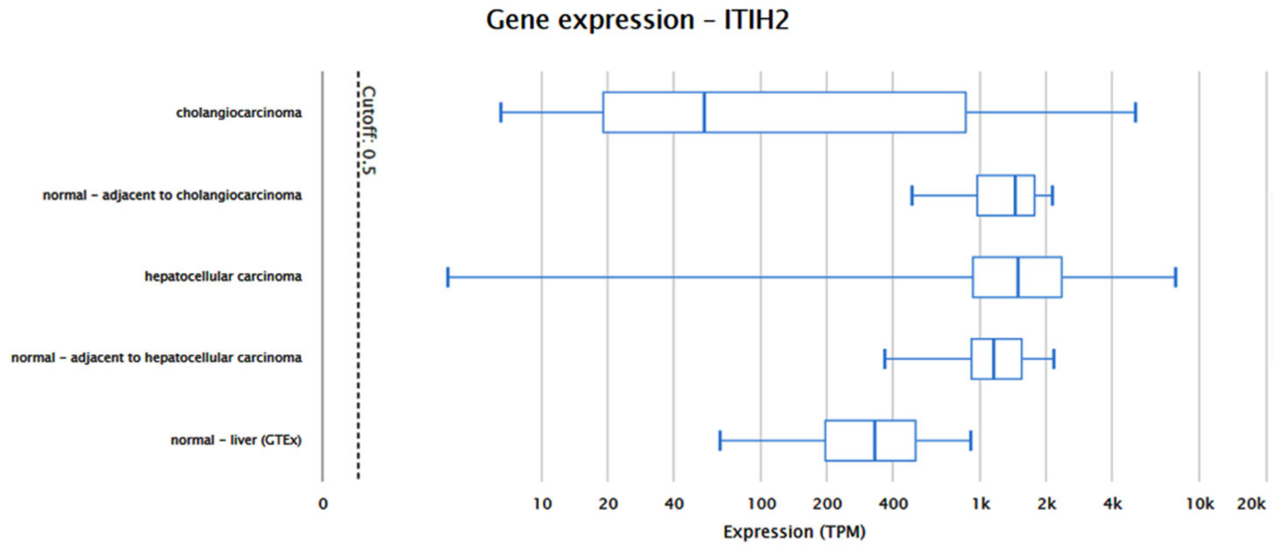

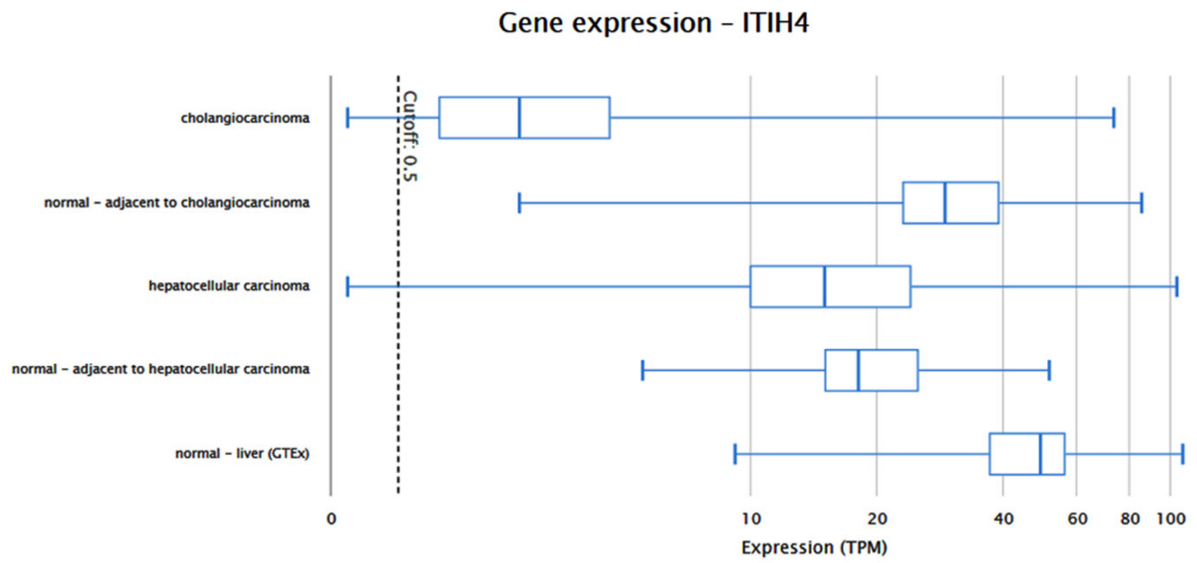

Gene expression - ITIH4
cholangiocarcinoma
normal - adjacent to cholangiocarcinoma
hepatocellular carcinoma
normal - adjacent to hepatocellular carcinoma
normal - liver (GTEx)
Cutoff: 0.5
0
10
20
40
60
80
100
Expression (TPM)

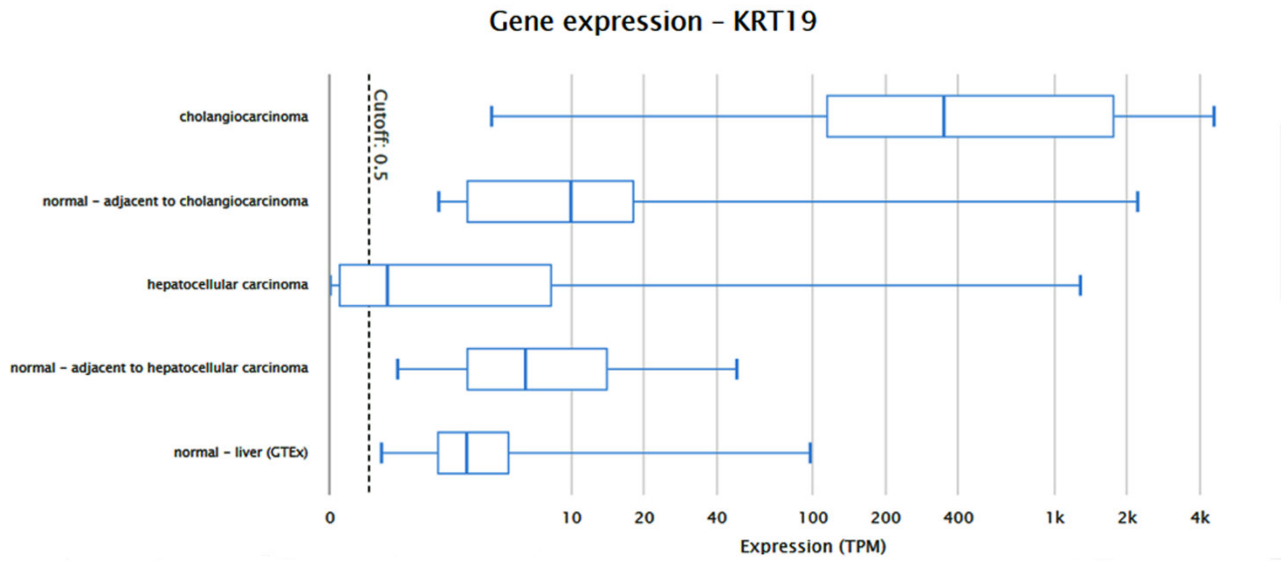

Gene expression - KRT19
cholangiocarcinoma
normal - adjacent to cholangiocarcinoma
hepatocellular carcinoma
normal - adjacent to hepatocellular carcinoma
normal - liver (GTEx)
Cutoff: 0.5
0
10
20
40
100
200
400
1k
2k
4k
Expression (TPM)

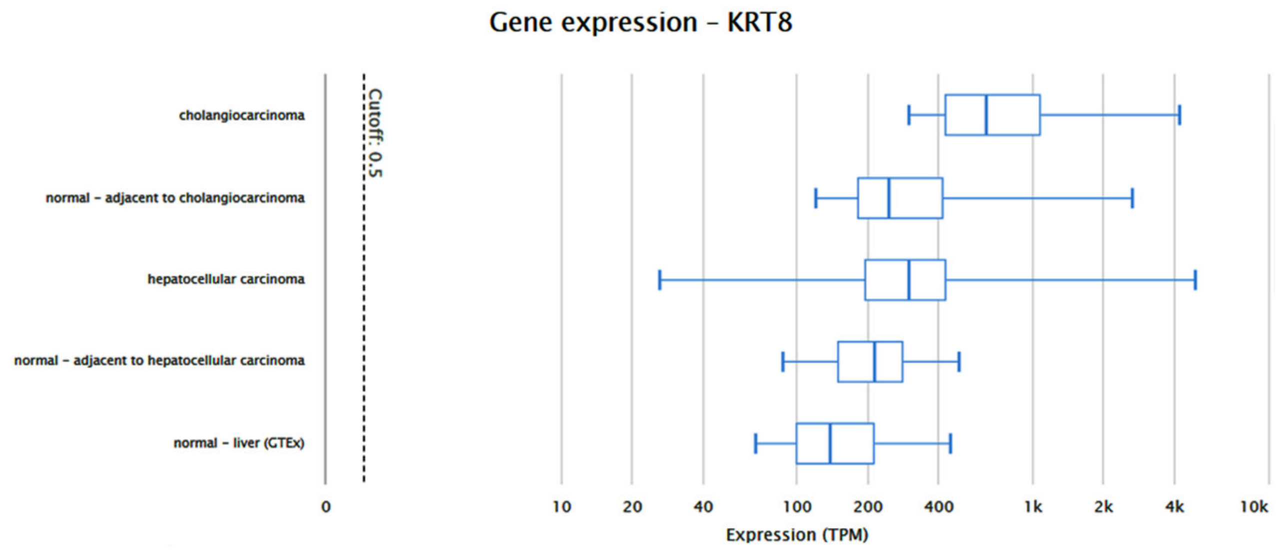

Gene expression - KRT8
cholangiocarcinoma
normal - adjacent to cholangiocarcinoma
hepatocellular carcinoma
normal - adjacent to hepatocellular carcinoma
normal - liver (GTEx)
Cutoff: 0.5
0
10
20
40
100
200
400
1k
2k
4k
10k
Expression (TPM)

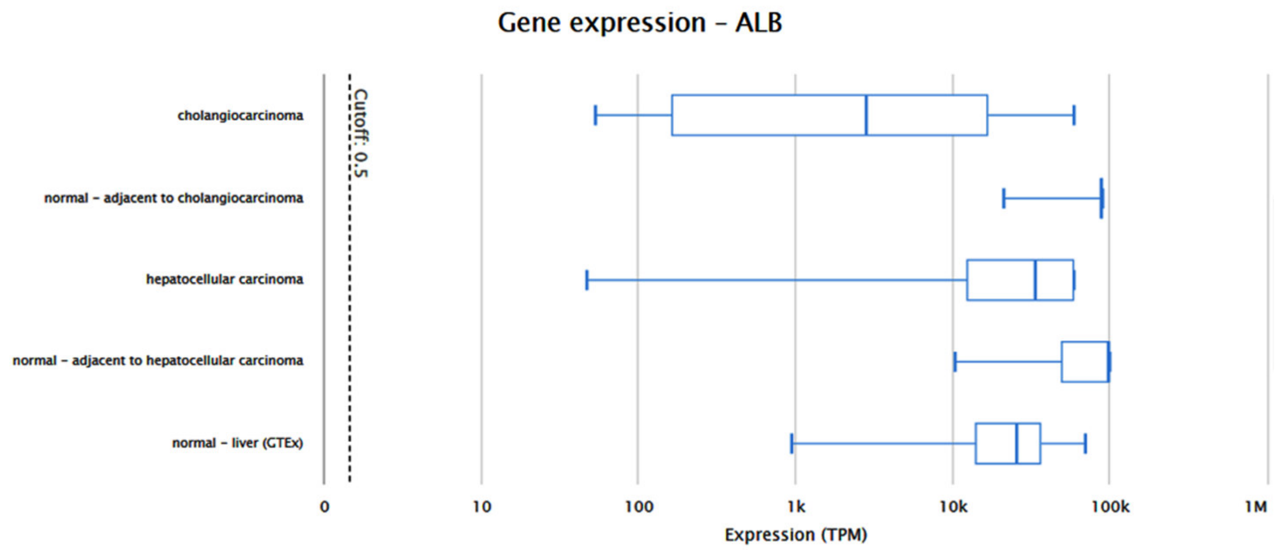

Gene expression - ALB
cholangiocarcinoma
normal - adjacent to cholangiocarcinoma
hepatocellular carcinoma
normal - adjacent to hepatocellular carcinoma
normal - liver (GTEx)
Cutoff: 0.5
0
10
100
1k
10k
100k
1M
Expression (TPM)

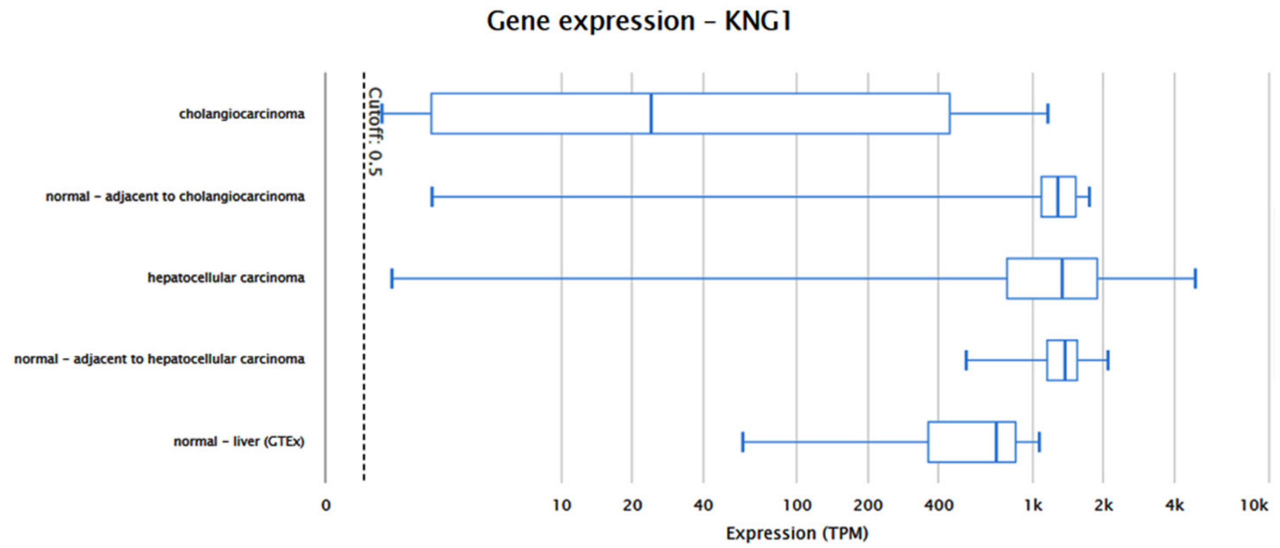

Gene expression - KNG1
cholangiocarcinoma
normal - adjacent to cholangiocarcinoma
hepatocellular carcinoma
normal - adjacent to hepatocellular carcinoma
normal - liver (GTEx)
Cutoff: 0.5
0
10
20
40
100
200
400
1k
2k
4k
10k
Expression (TPM)

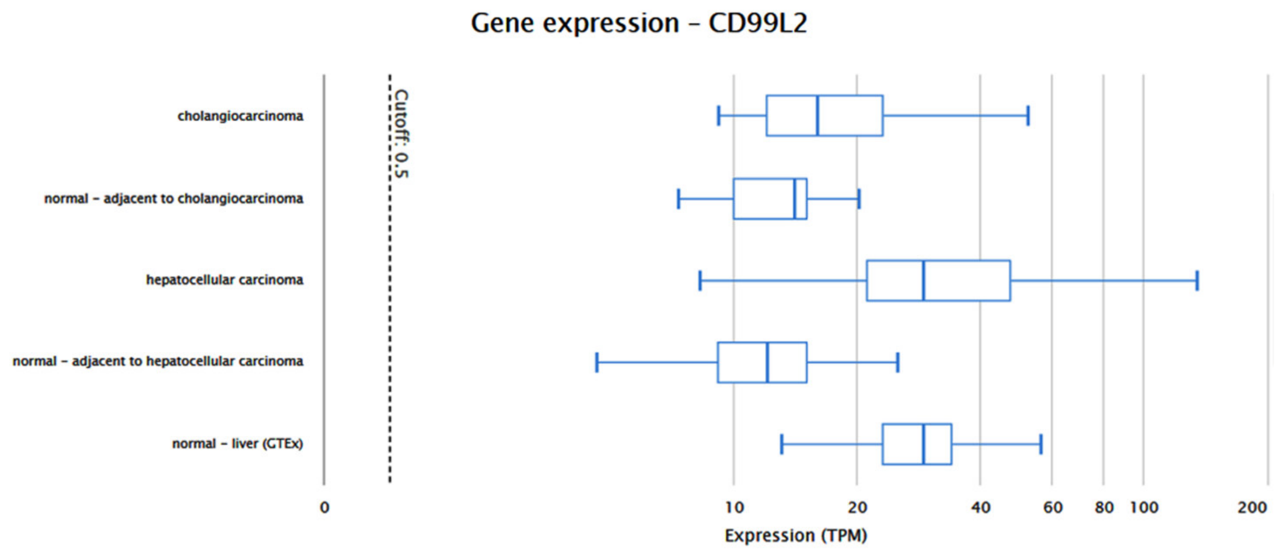

Gene expression - CD99L2
cholangiocarcinoma
normal - adjacent to cholangiocarcinoma
hepatocellular carcinoma
normal - adjacent to hepatocellular carcinoma
normal - liver (GTEx)
Cutoff: 0.5
0
10
20
40
60
80
100
200
Expression (TPM)

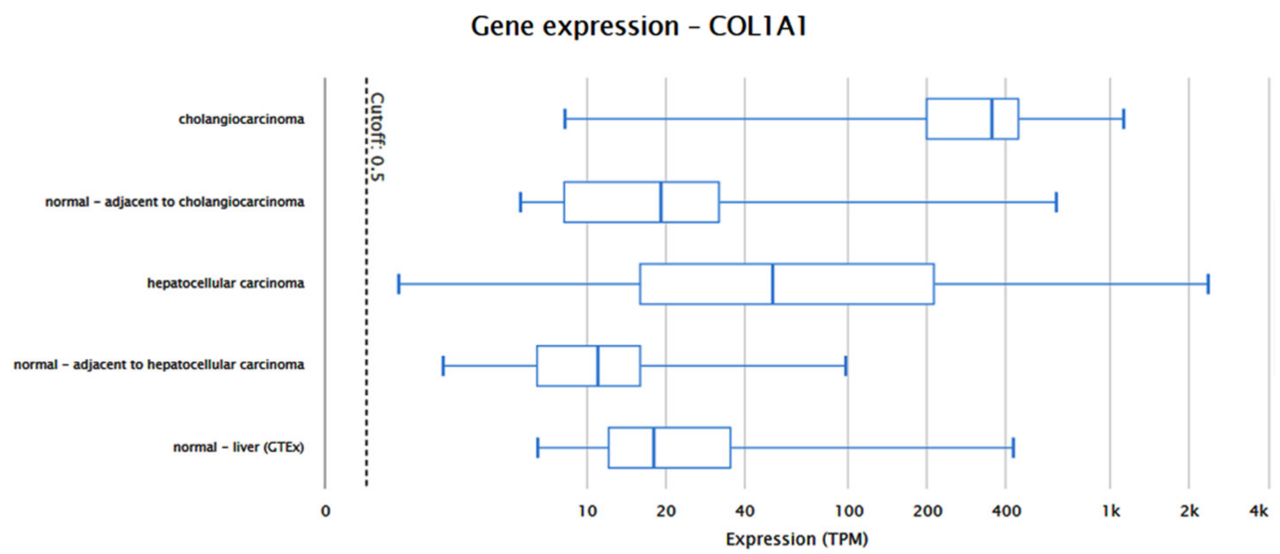
Gene expression – COL1A1
cholangiocarcinoma
normal – adjacent to cholangiocarcinoma
hepatocellular carcinoma
normal – adjacent to hepatocellular carcinoma
normal – liver (GTEx)
Cutoff: 0.5
0
10
20
40
100
200
400
1k
2k
4k
Expression (TPM)

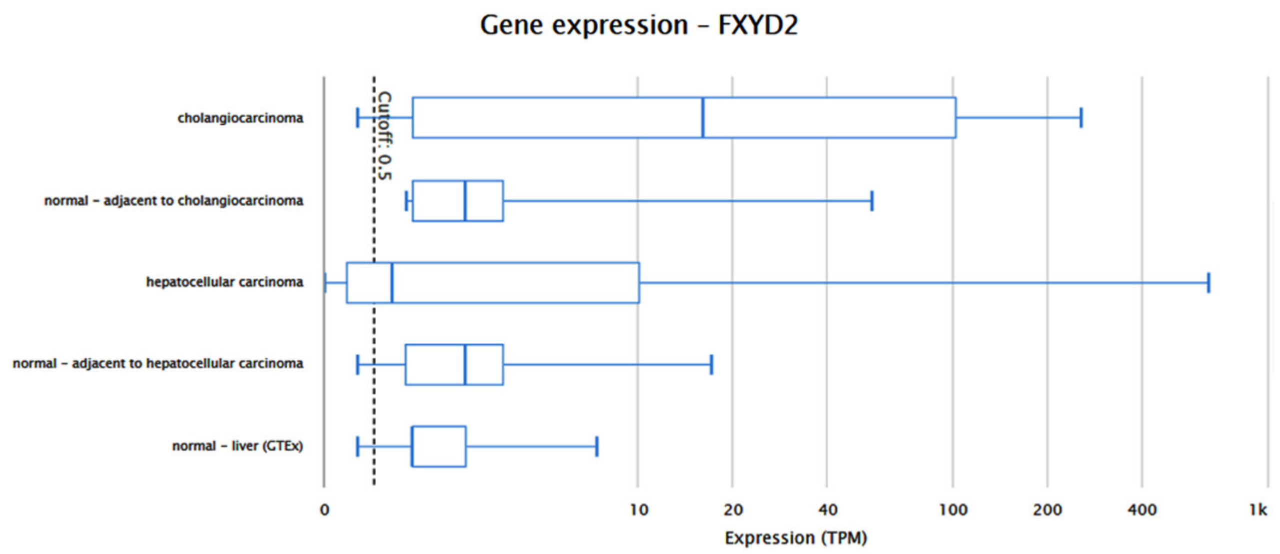
Gene expression – FXYD2
cholangiocarcinoma
normal – adjacent to cholangiocarcinoma
hepatocellular carcinoma
normal – adjacent to hepatocellular carcinoma
normal – liver (GTEx)
Cutoff: 0.5
0
10
20
40
100
200
400
1k
Expression (TPM)

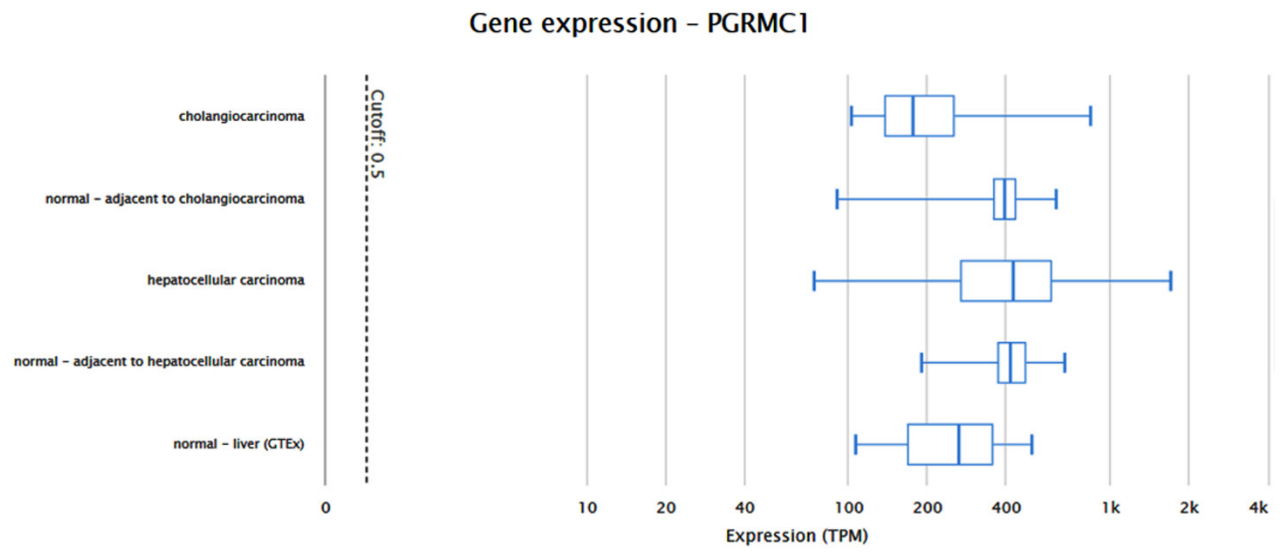
Gene expression – PGRMC1
cholangiocarcinoma
normal – adjacent to cholangiocarcinoma
hepatocellular carcinoma
normal – adjacent to hepatocellular carcinoma
normal – liver (GTEx)
Cutoff: 0.5
0
10
20
40
100
200
400
1k
2k
4k
Expression (TPM)

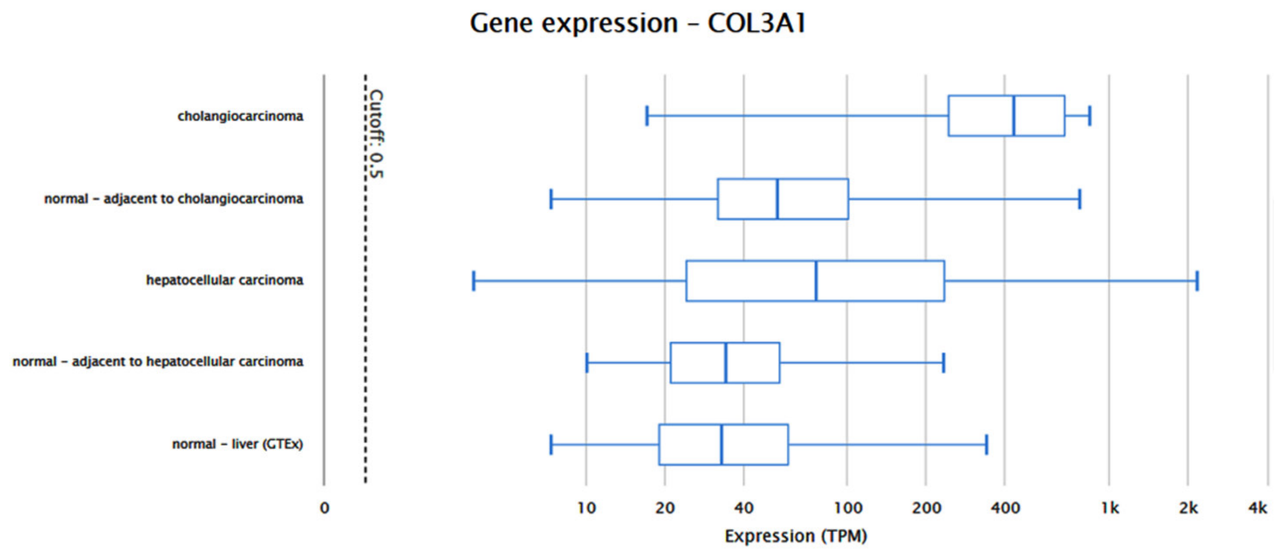
Gene expression – COL3A1
cholangiocarcinoma
normal – adjacent to cholangiocarcinoma
hepatocellular carcinoma
normal – adjacent to hepatocellular carcinoma
normal – liver (GTEx)
Cutoff: 0.5
0
10
20
40
100
200
400
1k
2k
4k
Expression (TPM)

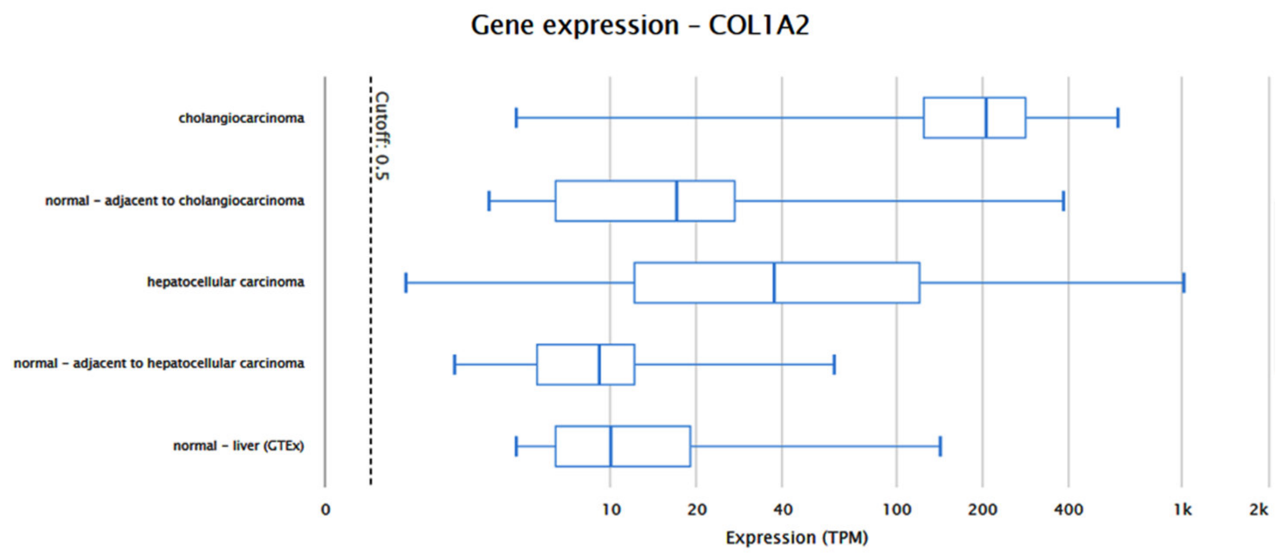
Gene expression – COL1A2
cholangiocarcinoma
normal – adjacent to cholangiocarcinoma
hepatocellular carcinoma
normal – adjacent to hepatocellular carcinoma
normal – liver (GTEx)
Cutoff: 0.5
0
10
20
40
100
200
400
1k
2k
Expression (TPM)

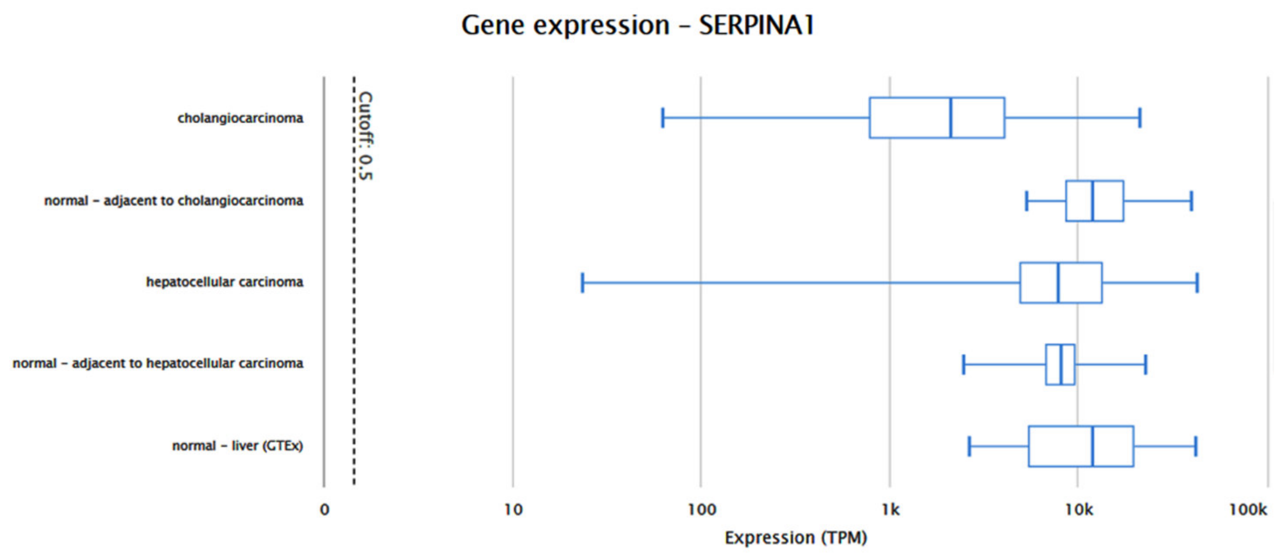
Gene expression – SERPINA1
cholangiocarcinoma
normal – adjacent to cholangiocarcinoma
hepatocellular carcinoma
normal – adjacent to hepatocellular carcinoma
normal – liver (GTEx)
Cutoff: 0.5
0
10
100
1k
10k
100k
Expression (TPM)

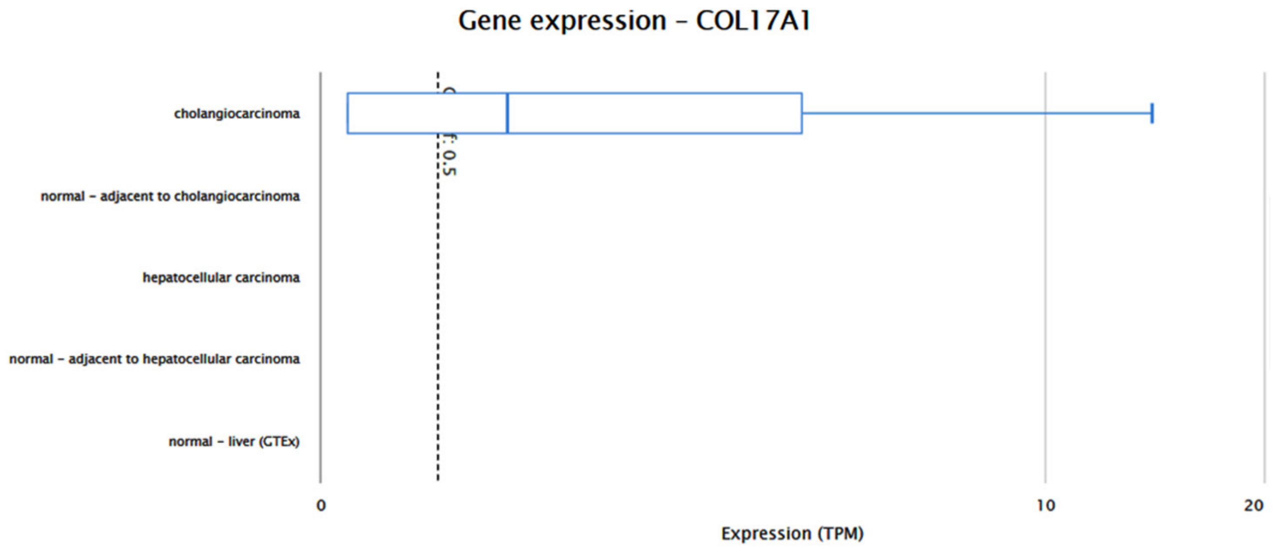
Gene expression - COL17A1
cholangiocarcinoma
normal - adjacent to cholangiocarcinoma
hepatocellular carcinoma
normal - adjacent to hepatocellular carcinoma
normal - liver (GTEx)
0
10
20
Expression (TPM)

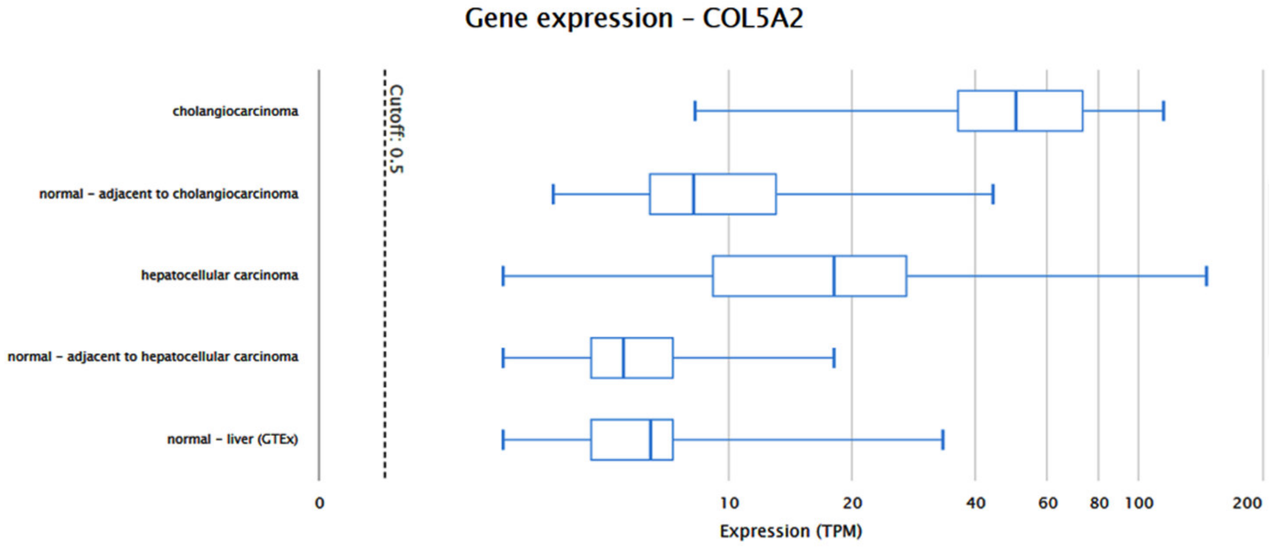
Gene expression - COL5A2
cholangiocarcinoma
normal - adjacent to cholangiocarcinoma
hepatocellular carcinoma
normal - adjacent to hepatocellular carcinoma
normal - liver (GTEx)
Cutoff: 0.5
0
10
20
40
60
80
100
200
Expression (TPM)

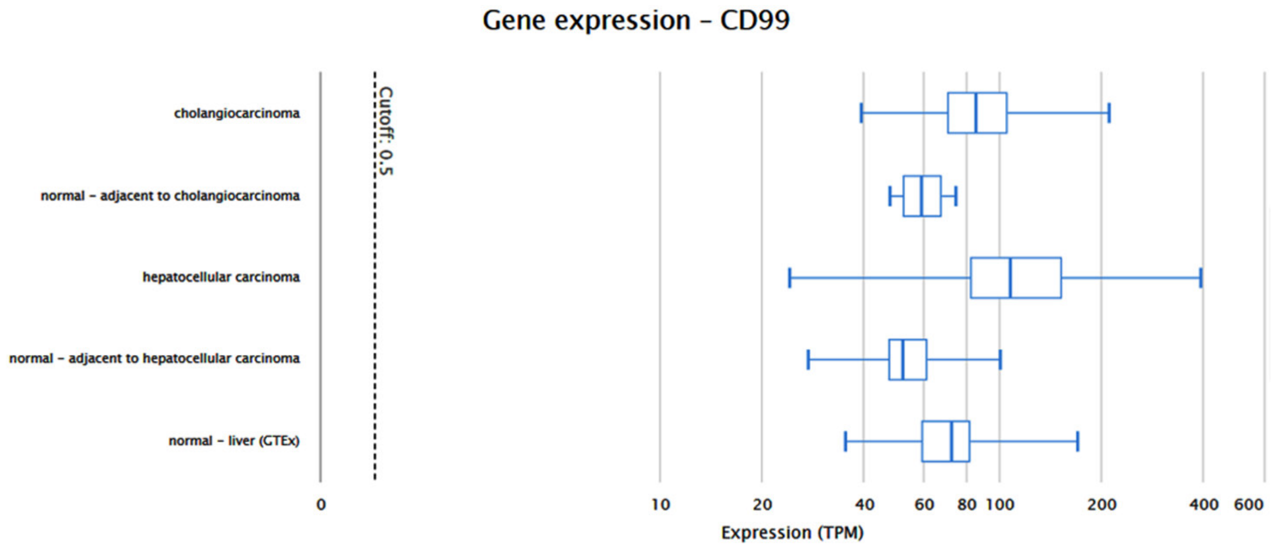
Gene expression - CD99
cholangiocarcinoma
normal - adjacent to cholangiocarcinoma
hepatocellular carcinoma
normal - adjacent to hepatocellular carcinoma
normal - liver (GTEx)
Cutoff: 0.5
0
10
20
40
60
80
100
200
400
600
Expression (TPM)

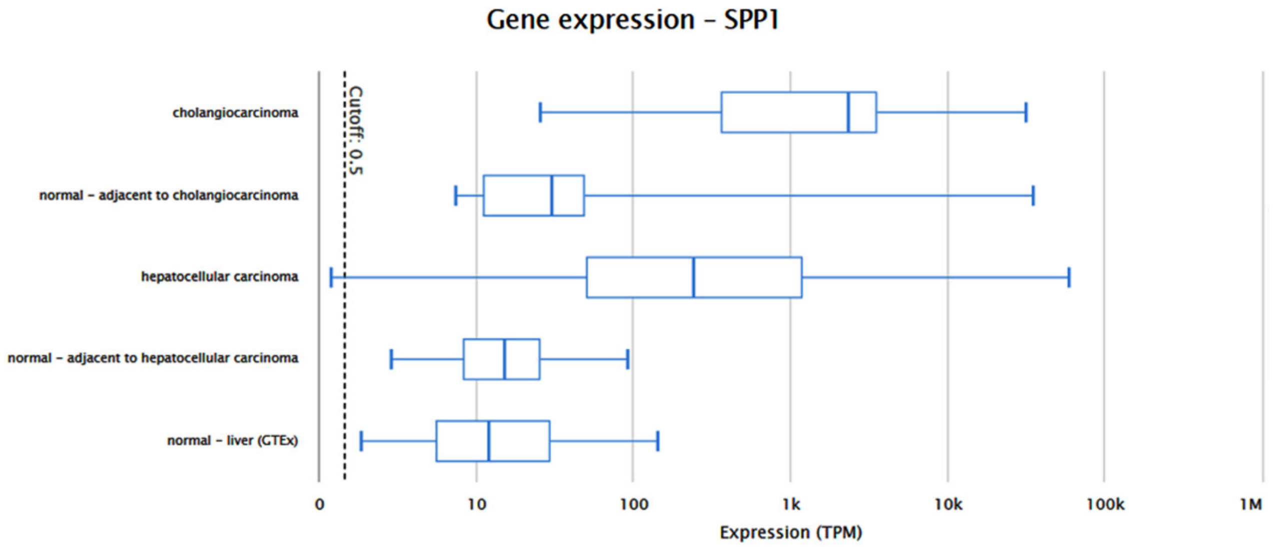
Gene expression - SPP1
cholangiocarcinoma
normal - adjacent to cholangiocarcinoma
hepatocellular carcinoma
normal - adjacent to hepatocellular carcinoma
normal - liver (GTEx)
Cutoff: 0.5
0
10
100
1k
10k
100k
1M
Expression (TPM)

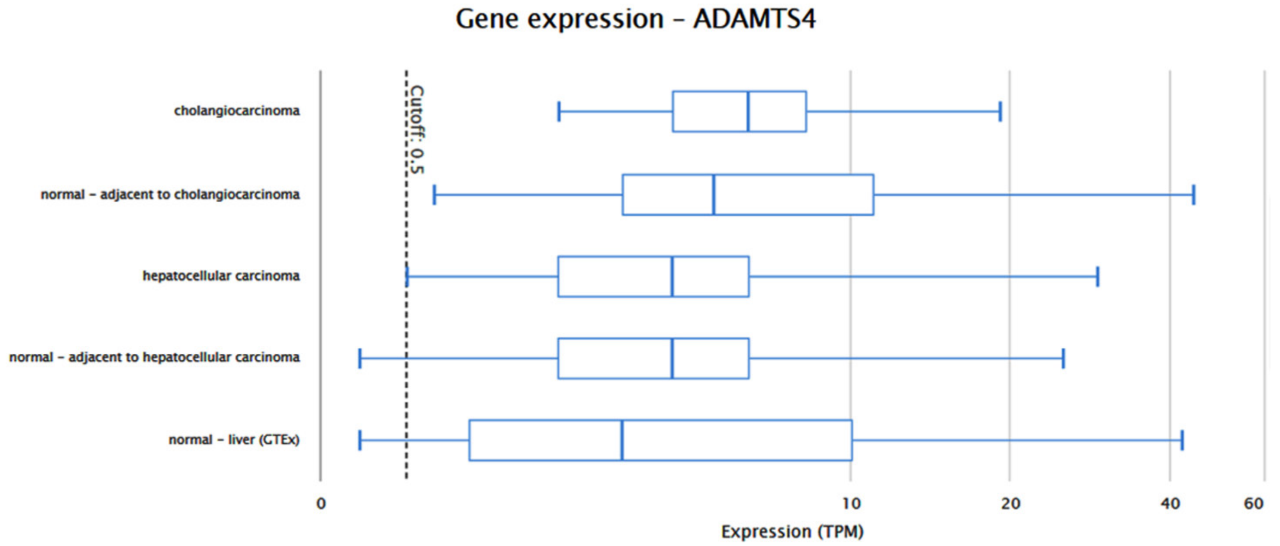
Gene expression - ADAMTS4
cholangiocarcinoma
normal - adjacent to cholangiocarcinoma
hepatocellular carcinoma
normal - adjacent to hepatocellular carcinoma
normal - liver (GTEx)
Cutoff: 0.5
0
10
20
40
60
Expression (TPM)

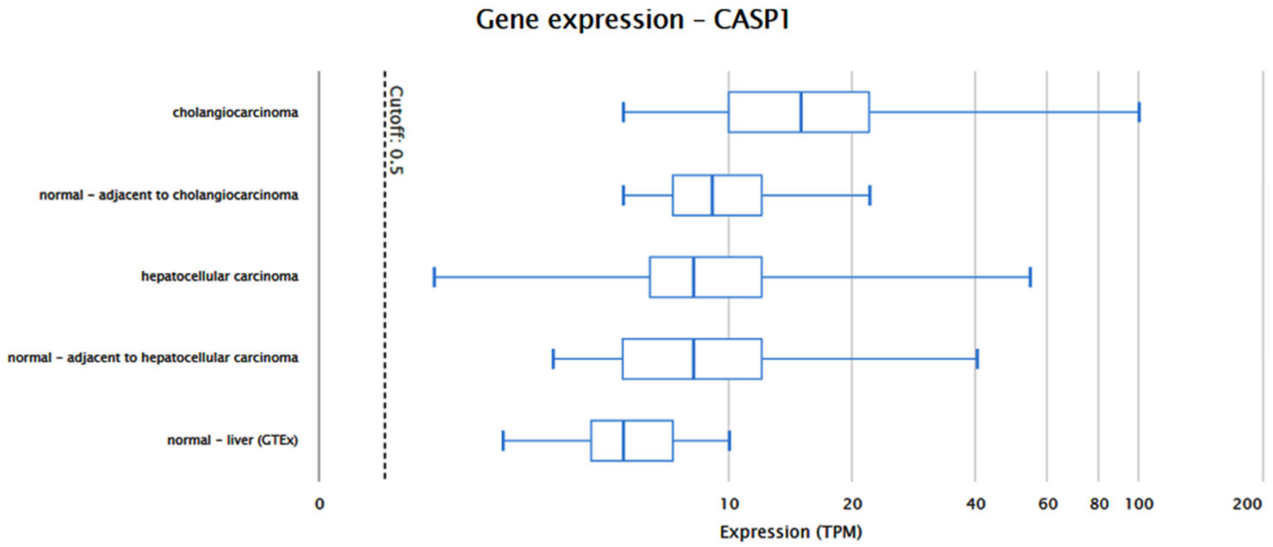
Gene expression - CASP1
cholangiocarcinoma
normal - adjacent to cholangiocarcinoma
hepatocellular carcinoma
normal - adjacent to hepatocellular carcinoma
normal - liver (GTEx)
Cutoff: 0.5
0
10
20
40
60
80
100
200
Expression (TPM)

Table S4. Physiological and cancer-specific pathological implications of the parental proteins from which the bile peptide markers for CCA diagnosis by bile proteome analysis are derived.

Protein	Characteristic physiological functions*	Link to cancer pathology	Relation to CCA pathology	Peptide marker	Peptide marker specific information
14-3-3 ζ/δ (YWHAZ)	Cell survival [1], autophagy [2], cell stress protection [2, 3].	EMT induction [4], tumor cell proliferation [5], chemoresistence [6].	Mediator of EMT, metastasis and invasion [4], prognostic marker for intrahepatic CCA [7], target for chemosensitivity enhancement [8].	28-SVTEQGAELSNEER-41	Release by trypsin 1 cleavage [9], Identification in urine during allograft kidney injury [10], epitope for HLA-A MHC I receptors in rats [11], inhibition of T cell migration [12].
Actin, cytoplasmic 1 (ACTB)	Cellular structure and cell motility [13], gene expression, cell division and proliferation [14].	Actin cytoskeleton reorganization during EMT [15], NO formation and platelet signaling [16].	Secreted by the CCA cell line huCCA-1 [17].	92-NELRVAPEEHPV-103<br>179-DLAGRDLTDYLm-190	Elevation of 92-NELRVAPEEHPV-103 in lung lavage fluid during bronchiolitis obliterans syndrome [18].
F-actin-capping protein subunit β (CAPZB)	Actin filament depolymerization and capping promoting cell motility [19].	Overexpression in tissue specimens of epithelioid sarcoma and association with cell growth and motility [20].	Upregulated in the membrane and cytosole of the human CCA cell line huCCA-1 [21].	76-SNKYDPPLEDGAMPS-90	Elevated in lung lavage fluid during bronchiolitis obliterans syndrome [18].
Hemoglobin subunit α and β (HBA1 and HBB)	Oxygen transport, heme binding, control of acid-base equilibrium	Peroxidase activity during inflammation and oxidative stress, and induction of cytotoxicity to macrophages by hemoglobin aggregate formation [22], hemoglobin release by cancer induced hemolysis [23].	Not systematically investigated, anemia is associated with CCA	**HBA1**:<br>27-AEALERmFL-35<br>33-MFLSFPTTK-41<br>63-VADALTNAVAHVDDMPNALSALSDLHAH-90<br>**HBB**:<br>2-VHLTPEEKSAVTA-14<br>43-FESFGDLSTPDAVmGNPK-60<br>67-KVLGAFSDGLA-77	Identification of 33-MFLSFPTTK-41 and 63-VADALTNAVAHVDDMPNALSALSDL HAH-90 as components of circulating exosomes in lung cancer [24], identification of 33-MFLSFPTTK-41 and 43-FESFGDLSTPDAVmGNPK-60 as injury marker in urine after kidney allograft transplantation [10], elevation of 2-VHLTPEEKSAVTA-14, 27-AEALERmFL-35 and 43-FESFGDLSTPDAVmGNPK-60 in lung lavage fluid during bronchiolitis obliterans syndrome [18].
Inter-α-trypsin inhibitor heavy chain H2 (ITIH2)	Plasma protease inhibitor activity [25], hyaluron carrier and binding mediator [26], contribution to extracellular matrix stability [27].	Tumor invasion and metastasis suppressor with loss of expression in invasive tumors [25, 28].	Reduced serum levels in CCA compared to benign biliary tract diseased patients [29].	628-LVIENEAGDER-638	-----
Inter-α-trypsin inhibitor heavy chain H4 (ITIH4)	Anti-inflammatory type II acute phase protein [30], formation of hyaluronan complexes [31], potentially involved in liver development and regeneration [32].	Prevention of tumor metastasis [33].	Upregulated in bile of malignant (CCA and pancreatic cancer) towards benign (mostly PSC) strictures [34].	381-ILLTDGDPTVGETNPR-396	Elevated in salvia of oral cancer patients [35].
Keratin, type I cytoskeletal 19 (KRT19)	Cytoskeletal component in epithelial cells [36], actin cytoskeletal organization [37].	Histology marker for adenocarcinomas and squamous cell carcinomas [38, 39].	Differentiation marker for CCA ($KRT19^{+}$) and HCC ($KRT19^{-}$) [40], KRT19 fragment CYFRA21-1 is a prognostic marker for intrahepatic CCA [41], reduced expression of KRT19 on CCA cell lines during EMT [42].	139-DKILGATIENS-149	-----

Protein	Characteristic physiological functions*	Link to cancer pathology	Relation to CCA pathology	Peptide marker	Peptide marker specific information
Keratin, type II cytoskeletal 8 (KRT8)	Cytoskeletal component in epithelial cells [43, 44], hepatobiliary epithelial marker with strongest expression in mature polarized cells.	Marker for epithelial tumors [45], reduced expression during EMT [46].	Secreted by the CCA cell line huCCA-1 [17], reduced expression in a sarcomatoid CCA cell line [47].	149-RQLETLGQEK-158<br>466-IETRDGKLVSESSDVLPK-483	Identification of 149-RQLETLGQEK-158 in the human fetal liver proteome [48].
Serum albumin (ALB)	Maintenance of oncotic pressure, transport of metabolites, antioxidant.	Immune modulation due to binding of pathogen and danger associated molecules and inflammatory mediators [49], evidence for intracellular blocking of inflammatory signaling pathways after endocytic internalization [50].	Reduced levels in intrahepatic CCA tumor tissue samples [51], levels > 3 g/dL are an independent predictor of overall survival in hilar CCA [52].	173-FYAPELLFFAK-183	Component of circulating exosomes in lung cancer[24], identification in urine during allograft kidney injury[10].

* According to the Uniprot knowledge database unless no other reference is given

Abbreviations: CCA, cholangiocarcinoma; EMT, epithelial–mesenchymal transition; HCC, hepatocellular carcinoma.

Table S5. Physiological and cancer-specific pathological implications of the parental proteins from which the urine peptide markers for CCA diagnosis by urine proteome analysis are derived.

Protein	Characteristic physiological functions*	Link to cancer pathology	Relation to CCA pathology	Peptide marker	Peptide marker specific information
Uromodulin (UMOD)	Salt and water retention, urinary host defense and protection against stone formation in the kidney [53], renal clearance of circulating cytokines [54], regulation of granulopoiesis via proximal epithelial activation of the IL-23/IL-17 axis [55].	Protection from kidney injury by inhibition of inflammation [56], immunomodulation of blood cells [57], expression under control of the cancer-associated transcriptional factor HNF1B [58, 59].	-----	589-SGSVIDQSR-597	Identification in human urine [60], differential urinary expression between muscle-invasive bladder cancer versus normal [61].
Kininogen-1 (KNG1)	Thiol protease inhibition [62], inhibition of endothelial cell proliferation and migration, angiogenesis and apoptosis [63], precursor of proinflammatory vasodilator kinins [64].	Serum biomarker for advanced colorectal adenoma and colorectal cancer [65].	Increased biliary expression in malignant versus benign strictures [34].	62-ATKTVGSDTF-71	Differential urinary expression during inflammatory bowel disease [66], cystic kidney disease [67] and heart failure [68].
CD99 antigen-like protein 2 (CD99L2)	Cell surface protein mediating intercellular adhesion and leukocyte extravasation through the endothelial basement membrane [69], enhanced surface expression by interaction with CD99 [70], Integrin β1-mediated downregulation inhibiting leukocyte transmigration [71].	Marker of a mesenchymal phenotype during EMT [72].	-----	26-DFDDFNLED-34	Identification in human urine [73], decreased urinary levels in cystic kidney disease [67].

Protein	Characteristic physiological functions*	Link to cancer pathology	Relation to CCA pathology	Peptide marker	Peptide marker specific information
Collagen α-1(I) (COL1A1)	Structural component of the extracellular matrix generated mainly from fibroblasts, binding of platelet-derived growth factor (PDGF) which is a potent mitogen for mesenchymal cells [74].	Overexpression in various cancer types promoting metastasis [75] and apoptosis inhibition in tumor cells [76].	Indicative for the presence of cancer-associated fibroblasts in the CCA tumor stroma [77]	222-PpGPpGKNGDDGEAGKP-238<br>288-EpGSpGENGApGQMGPR-304<br>543-SpGSPGPDGKTGPpGPAG-560<br>550-DGKTGpPGPAGQDGRPGPpGppG-572<br>613-DGEAGAQGPpGPA-625<br>657-KpGEQGVpGDLG-668<br>780-DKGESGpSGpAGpTGARGApGDRGEpGppG-809<br>918-RpGEVGPpGPpGP-930<br>1007-GPpGESGREGApG-1019<br>1021-EGSpGRDGSpGAKGDRGETGPA-1042	Differential urinary expression in chronic kidney [67, 78, 79], cardiovascular [68] and chronic inflammatory [66] diseases.
Collagen α-1(II) (COL2A1)	Main structural component of cartilaginous tissues [80], PDGF binding [74], proteoglycan binding [81].	Increased expression indicates tumor recurrence in high-grade serous ovarian cancer [82].	Among the genes that are associated with lymph node metastasis and perineural invasion of CCA [83].	1212-PGNPGPPGPPGPpGpGIDmSAFAG-1225	-----
Collagen α-1(III) (COL3A1)	Main structural component of reticular fibers in soft tissues such as liver along with type I collagen [84], binding to platelets [85].	Increased expression in bladder cancer with association to poor prognosis and focal adhesion [86].	Indicative for the presence of cancer-associated fibroblasts in the CCA tumor stroma [77].	1084-ApGPQGPRGDkGETGERG-1101	Differential urinary expression during rheumatoid arthritis [66].
Collagen α-2(I) (COL1A2)	Extracellular matrix structural component, PDGF binding [74].	Increased expression and prognostic marker in gastric cancer [87].	Altered lysine hydroxylation in a Pten$^{-/-}$ mouse model representing a mixed phenotype of HCC and CCA with impact on matrix remodeling and stiffening [88].	136-GPpGKAGEDGHpGKPGRpGERG-157<br>188-LkGQpGApGVKGEpGApGENGTPGQTGARG-217<br>188-LkGQpGApGVkGEpGApGENGTpGQTGARG-217<br>189-kGQpGApGVkGEpGApGENGTpGQTGARG-217	Increased urinary levels of 136-GPpGKAGEDGHpGKPGRpGERG-157, 188-LkGQpGApGVKGEpGApGENGTPGQTGARG-217 and 189-kGQpGApGVkGEpGAp GENGTpGQTGARG-217 in lupus nephritis [79], differential expression of 136-GPpGKAGEDGHpGKPGRpGERG-157 in muscle-invasive bladder cancer [61], differential expression of 188-LkGQpGApG VkGEpGApGENGTpGQTGARG-217 in chronic kidney disease versus healthy controls [89].
Collagen α-2(V) (COL5A2)	Key determinant in the assembly of tissue matrices with binding affinity to DNA, heparan sulfate, thrombospondin, heparin, and insulin.	Increased expression in muscle-invasive bladder cancer associated with poor prognosis and tumor invasion [90].	-----	833-PGSRGENGPTGAVGFAGPQGPDGQPGVKGEP-863	Differential urinary expression during rheumatoid arthritis [66].
Collagen α-6(VI) (COL6A6)	Component of the basal lamina of epithelial cells potentially involved in the regulation of epithelial cell-fibronectin interactions [91].	Correlation of high tissue expression levels with early tumor stages in breast cancer [92].	Upregulation in a Pten$^{-/-}$ mouse model representing a mixed phenotype of HCC and CCA [88].	1701-GLpGEmGSpGEPG-1713	-----
Collagen α-1(XVII) (COL17A1)	Structural component of hemidesmosomes mediating adhesion of keratinocytes to the underlying epidermal basement membrane [93].	Overexpression in epithelial cancers due to aberrant epigenetic control promoting tumor invasion [94].	Elevated mRNA expression in cholangiocarcinoma tissue [95].	599-PGpQGpKGQKGSVGDpGMEGPmGQRGREG-627<br>628-PMGpRGEAGppGSGEKGERGAAGEPGp-654	-----

Protein	Characteristic physiological functions*	Link to cancer pathology	Relation to CCA pathology	Peptide marker	Peptide marker specific information
Na^+/K^+-ATPase γ subunit (FXYD2)	Modulation of sodium ATPase activity [96].	Increased expression in ovarian clear cell carcinoma under control of the transcriptional factor HNF1B [97].	Differential expression in CCA cells [98].	3-GLSMDGGGSPKGDVDP-18	Reduced urinary expression in chronic kidney disease [89] and overt heart failure [99].
Membrane-associated progesterone receptor component 1 (PGRMC1)	Compoment of a progesterone-binding membrane complex [100], progesterone binding [101], many other cellular functions like heme and cytochrome P450 binding, autophagy and apoptosis induction [100].	Regulation of cancer proliferation by EGF receptor and cytochrome P450 interaction [102], differential cell surface expression in various tumors like breast and bladder cancer [103, 104], negative regulation of hepatocarcinogenesis [105].	----	54-SGDSDDDEPPPLPRL-68	Reduced urinary expression in chronic kidney disease [89], cardiovascular disease [106], inflammatory bowel disease [66] and muscle-invasive bladder cancer [107].
α-1-antitrypsin (SERPINA1)	Acute phase protein with anti-inflammatory and immunoregulatory functions [108], serine protease inhibition including neutrophil elastase, plasmin, thrombin, kallikreins, matripase, caspase-3 [109], induction of IL-1 receptor antagonist expression [110].	Association of α-1-antitrypsin deficiency with increased risk of liver cirrhosis and hepatocellular carcinoma [111], lung disease [112] and ANCA-associated vasculitis [113].	Increased serum levels as marker for early CCA diagnosis [114], increased oxidation due to oxidative stress in CCA [115].	25-EDPQGDAAQKTDTSHHDQDHP-45	Reduced urinary expression during preclinical cardiac dysfunction before heart failure [68], increase in urinary expression by inhibition of the sodium/glucose cotransporter 2 with empagliflozin in patients with diabetic kidney disease [116].
Interleukin-1 receptor accessory protein-like 1 (IL1RAPL1)	Member of the TOLL/IL-1 receptor familiy [117], IL-38 binding [118], negative regulation of calcium-dependent exocytosis [119], evidence for selective activation of JNK [120], identification in exosomes of mesenchymal stem cells [121].	Upregulation upon γδ T cell activation [122], inhibition of cytokine release by macrophages upon antagonizing IL-38 binding [118].	Predicted as target for miR-10a-5p upregulation in human CCA cell lines [123].	74-LMWYKSSGPGDFEEPIAFDGSRmSKEED-101	----
CD99 antigen (CD99)	Cell surface protein involved in cell migration, adhesion, diapedesis, death and differentiation of leukocytes [69, 124], inhibition of cell-ECM adhesion by integrin 1-β suppression [125].	Cancer type specific marker for neuroectodermal and non-small cell lung carcinomas [126, 127], increased expression in inflammatory diseases [128].	Decreased expression in gastric and gallbladder carcinomas due to promoter methylation, loss of heterozygosity and transcription factor down-regulation associated with unfavorable prognosis [129, 130].	97-DGVSGGEGKGGSDGGGSHRKEGEEADAPGVIPGIVG-132	Differential expression in urine between muscle-invasive bladder cancer versus normal [61].
Osteopontin (SPP1)	Bone mineralisation [131], matricellular ECM component [132], CD44-dependent chemotaxis [133], mediation of type 1 immunity [134].	Overexpression in various cancer types with protumorigenic and premetastatic activity [135], contribution to scarring and liver fibrosis via TGF-β [136], immune checkpoint by CD44 binding promoting tumor invasion [137, 138].	Elevated serum levels in patients with CCA compared to healthy controls and patients with PSC [139], association of elevated serum levels with shorter overall survival and high probability of tumor relapse after curative resection in patients with intrahepatic CCA [140].	194-ESEELNGAYKAIPVAQDLNAPSDWDSRGKDSYETSQL-230	-----

* According to the Uniprot knowledge database unless no other reference is given

Abbreviations: CCA; cholangiocarcinoma; EMT; epithelial–mesenchymal transition; HCC, hepatocellular carcinoma.

Supplementary references – Literature review

1. Liang R, Chen XQ, Bai QX, Wang Z, Zhang T, Yang L, et al. Increased 14-3-3ζ expression in the multidrug-resistant leukemia cell line HL-60/VCR as compared to the parental line mediates cell growth and apoptosis in part through modification of gene expression.. Acta Haematol. 2014;132:177-86.
2. Weerasekara VK, Panek DJ, Broadbent DG, Mortenson JB, Mathis AD, Logan GN, et al. Metabolic-stress-induced rearrangement of the 14-3-3ζ interactome promotes autophagy via a ULK1- and AMPK-regulated 14-3-3ζ interaction with phosphorylated Atg9. Mol Cell Biol. 2014;34:4379-88.
3. Tang Y, Liu S, Li N, Guo W, Shi J, Yu H, et al. 14-3-3ζ promotes hepatocellular carcinoma venous metastasis by modulating hypoxia-inducible factor-1α. Oncotarget. 2016;7:15854-67.
4. Yang Y, Liu Y, He JC, Wang JM, Schemmer P, Ma CQ, et al. 14-3-3ζ and aPKC-ι synergistically facilitate epithelial-mesenchymal transition of cholangiocarcinoma via GSK-3β/Snail signaling pathway. Oncotarget. 2016;7:55191-210.
5. Nishimura Y, Komatsu S, Ichikawa D, Nagata H, Hirajima S, Takeshita H, et al. Overexpression of YWHAZ relates to tumor cell proliferation and malignant outcome of gastric carcinoma. Br J Cancer. 2013;108:1324-31.
6. Matta A, Siu KW, Ralhan R. 14-3-3 zeta as novel molecular target for cancer therapy. Expert Opin Ther Targets. 2012;16:515-23.
7. Zhang C, Liu LX, Dong ZR, Shi GM, Cai JB, Zhang PF, et al. Up-regulation of 14-3-3ζ expression in intrahepatic cholangiocarcinoma and its clinical implications. Tumour Biol. 2015;36:1781-9.
8. Kittirat Y, Techasen A, Thongchot S, Loilome W, Thanan R, Yongvanit P, et al. Suppression of 14-3-3ζ in cholangiocarcinoma cells inhibits proliferation through attenuated Akt activity, enhancing chemosensitivity to gemcitabine. Oncol Lett. 2018;15:347-53.
9. Schilling O, Overall CM. Proteome-derived, database-searchable peptide libraries for identifying protease cleavage sites. Nat Biotechnol. 2008;26:685-94.
10. Sigdel TK, Salomonis N, Nicora CD, Ryu S, He J, Dinh V, et al. The identification of novel potential injury mechanisms and candidate biomarkers in renal allograft rejection by quantitative proteomics. Mol Cell Proteomics. 2014;13:621-31.
11. Barnea E, Melamed Kadosh D, Haimovich Y, Satumtira N, Dorris ML, Nguyen MT, et al. The Human Leukocyte Antigen (HLA)-B27 Peptidome in Vivo, in Spondyloarthritis-susceptible HLA-B27 Transgenic Rats and the Effect of Erap1 Deletion. Mol Cell Proteomics. 2017;16:642-62.
12. Tripathy D, Clement SC, Schwenke DC, Banerji M, Bray GA, Buchanan TA, et al. Baseline adiponectin levels do not influence the response to pioglitazone in ACT NOW. Diabetes Care. 2014;37:1706-11.
13. Drazic A, Aksnes H, Marie M, Boczkowska M, Varland S, Timmerman E, et al. NAA80 is actin's N-terminal acetyltransferase and regulates cytoskeleton assembly and cell motility. Proc Natl Acad Sci U S A. 2018;115:4399-404.
14. Cuvertino S, Stuart HM, Chandler KE, Roberts NA, Armstrong R, Bernardini L, et al. ACTB Loss-of-Function Mutations Result in a Pleiotropic Developmental Disorder. Am J Hum Genet. 2017;101:1021-33.
15. Boland S, Boisvieux-Ulrich E, Houcine O, Baeza-Squiban A, Pouchelet M, Schoëvaërt D, Marano F. TGF beta 1 promotes actin cytoskeleton reorganization and migratory phenotype in epithelial tracheal cells in primary culture. J Cell Sci. 1996;109:2207-19.
16. Ji Y, Ferracci G, Warley A, Ward M, Leung KY, Samsuddin S, et al. beta-Actin regulates platelet nitric oxide synthase 3 activity through interaction with heat shock protein 90. Proc Natl Acad Sci U S A. 2007;104:8839-44.
17. Srisomsap C, Sawangareetrakul P, Subhasitanont P, Chokchaichamnankit D, Chiablaem K, Bhudhisawasdi V, et al. Proteomic studies of cholangiocarcinoma and hepatocellular carcinoma cell secretomes. J Biomed Biotechnol. 2010;2010:437143.
18. Stone MD, Harvey SB, Nelsestuen GL, Reilly C, Hertz MI, Wendt CH. Elevated peptides in lung lavage fluid associated with bronchiolitis obliterans syndrome. PLoS One. 2014;9:e84471.
19. Bai SW, Herrera-Abreu MT, Rohn JL, Racine V, Tajadura V, Suryavanshi N, et al. Identification and characterization of a set of conserved and new regulators of cytoskeletal organization, cell morphology and migration. BMC Biol. 2011;9:54.
20. Mukaihara K, Suehara Y, Kohsaka S, Kubota D, Toda-Ishii M, Akaike K, et al. Expression of F-actin-capping protein subunit beta, CAPZB, is associated with cell growth and motility in epithelioid sarcoma. BMC Cancer. 2016;16:206.
21. Srisomsap C, Subhasitanont P, Sawangareetrakul P, Chokchaichamnankit D, Ngiwsara L, Chiablaem K, Svasti J. Comparison of membrane-associated proteins in human cholangiocarcinoma and hepatocellular carcinoma cell lines. Proteomics Clin Appl. 2007;1:89-106.
22. Kapralov A, Vlasova II, Feng W, Maeda A, Walson K, Tyurin VA, et al. Peroxidase activity of hemoglobin-haptoglobin complexes: covalent aggregation and oxidative stress in plasma and macrophages. J Biol Chem. 2009;284:30395-407.
23. Altinoz MA, Elmaci I, Ince B, Ozpinar A, Sav AM. Hemoglobins, Hemorphins, and 11p15.5 Chromosomal Region in Cancer Biology and İmmunity with Special Emphasis for Brain Tumors. J Neurol Surg A Cent Eur Neurosurg. 2016;77:247-57.
24. Ueda K, Ishikawa N, Tatsuguchi A, Saichi N, Fujii R, Nakagawa H. Antibody-coupled monolithic silica microtips for highthroughput molecular profiling of circulating exosomes. Sci Rep. 2014;4:6232.
25. Hamm A, Veeck J, Bektas N, Wild PJ, Hartmann A, Heindrichs U, et al. Frequent expression loss of Inter-alpha-trypsin inhibitor heavy chain (ITIH) genes in multiple human solid tumors: a systematic expression analysis. BMC Cancer. 2008;8:25.
26. Huang L, Yoneda M, Kimata K. A serum-derived hyaluronan-associated protein (SHAP) is the heavy chain of the inter alpha-trypsin inhibitor. J Biol Chem. 1993;268:26725-30.
27. Bost F, Diarra-Mehrpour M, Martin JP. Inter-alpha-trypsin inhibitor proteoglycan family--a group of proteins binding and stabilizing the extracellular matrix. Eur J Biochem. 1998;252:339-46.
28. Paris S, Sesboüé R, Delpech B, Chauzy C, Thiberville L, Martin JP, et al. Inhibition of tumor growth and metastatic spreading by overexpression of inter-alpha-trypsin inhibitor family chains. Int J Cancer. 2002;97:615-20.
29. Janvilisri T, Leelawat K, Roytrakul S, Paemanee A, Tohtong R. Novel Serum Biomarkers to Differentiate Cholangiocarcinoma from Benign Biliary Tract Diseases Using a Proteomic Approach. Dis Markers. 2015;2015:105358.
30. Kashyap RS, Nayak AR, Deshpande PS, Kabra D, Purohit HJ, Taori GM, Daginawala HF. Inter-alpha-trypsin inhibitor heavy chain 4 is a novel marker of acute ischemic stroke. Clin Chim Acta. 2009;402:160-3.
31. Ivancic MM, Irving AA, Jonakin KG, Dove WF, Sussman MR. The concentrations of EGFR, LRG1, ITIH4, and F5 in serum correlate with the number of colonic adenomas in ApcPirc/+ rats. Cancer Prev Res (Phila). 2014;7:1160-9.
32. Bhanumathy CD, Tang Y, Monga SP, Katuri V, Cox JA, Mishra B, Mishra L. Itih-4, a serine protease inhibitor regulated in interleukin-6-dependent liver formation: role in liver development and regeneration. Dev Dyn. 2002;223:59-69.
33. Weidle UH, Birzele F, Tiefenthaler G. Potential of Protein-based Anti-metastatic Therapy with Serpins and Inter α-Trypsin Inhibitors. Cancer Genomics Proteomics. 2018;15:225-38.
34. Navaneethan U, Lourdusamy V, Gk Venkatesh P, Willard B, Sanaka MR, Parsi MA. Bile proteomics for differentiation of malignant from benign biliary strictures: a pilot study. Gastroenterol Rep (Oxf). 2015;3:136-43.
35. Xie H, Onsongo G, Popko J, de Jong EP, Cao J, Carlis JV, et al. Proteomics analysis of cells in whole saliva from oral cancer patients via value-added three-dimensional peptide fractionation and tandem mass spectrometry. Mol Cell Proteomics. 2008;7:486-98.
36. Moll R, Franke WW, Schiller DL, Geiger B, Krepler R. The catalog of human cytokeratins: patterns of expression in normal epithelia, tumors and cultured cells. Cell. 1982;31:11-24.
37. Eckert RL. Sequence of the human 40-kDa keratin reveals an unusual structure with very high sequence identity to the corresponding bovine keratin. Proc Natl Acad Sci U S A. 1988;85:1114-8.

38. Jain R, Fischer S, Serra S, Chetty R. The use of Cytokeratin 19 (CK19) immunohistochemistry in lesions of the pancreas, gastrointestinal tract, and liver. Appl Immunohistochem Mol Morphol. 2010;18:9-15.
39. Suo Z, Holm R, Nesland JM. Squamous cell carcinomas. An immunohistochemical study of cytokeratins and involucrin in primary and metastatic tumours. Histopathology. 1993;23:45-54.
40. Krishna M. Diagnosis of metastatic neoplasms: an immunohistochemical approach. Arch Pathol Lab Med. 2010;134:207-15.
41. Uenishi T, Yamazaki O, Tanaka H, Takemura S, Yamamoto T, Tanaka S, et al. Serum cytokeratin 19 fragment (CYFRA21-1) as a prognostic factor in intrahepatic cholangiocarcinoma. Ann Surg Oncol. 2008;15:583-9.
42. Techasen A, Loilome W, Namwat N, Dokduang H, Jongthawin J, Yongvanit P. Cytokines released from activated human macrophages induce epithelial mesenchymal transition markers of cholangiocarcinoma cells. Asian Pac J Cancer Prev. 2012;13 Suppl:115-8.
43. Owens DW, Wilson NJ, Hill AJ, Rugg EL, Porter RM, Hutcheson AM, et al. Human keratin 8 mutations that disturb filament assembly observed in inflammatory bowel disease patients. J Cell Sci. 2004;117(Pt 10):1989-99.
44. Casanova ML, Bravo A, Ramírez A, Morreale de Escobar G, Were F, Merlino G, et al. Exocrine pancreatic disorders in transsgenic mice expressing human keratin 8. J Clin Invest. 1999;103:1587-95.
45. Allard WJ, Matera J, Miller MC, Repollet M, Connelly MC, Rao C, et al. Tumor cells circulate in the peripheral blood of all major carcinomas but not in healthy subjects or patients with nonmalignant diseases. Clin Cancer Res. 2004;10:6897-904.
46. Knösel T, Emde V, Schlüns K, Schlag PM, Dietel M, Petersen I. Cytokeratin profiles identify diagnostic signatures in colorectal cancer using multiplex analysis of tissue microarrays. Cell Oncol. 2006;28:167-75.
47. Yoo HJ, Yun BR, Kwon JH, Ahn HS, Seol MA, Lee MJ, et al. Genetic and expression alterations in association with the sarcomatous change of cholangiocarcinoma cells. Exp Mol Med. 2009;41:102-15.
48. Ying W, Jiang Y, Guo L, Hao Y, Zhang Y, Wu S, et al. A dataset of human fetal liver proteome identified by subcellular fractionation and multiple protein separation and identification technology. Mol Cell Proteomics. 2006;5:1703-7.
49. Arroyo V, García-Martinez R, Salvatella X. Human serum albumin, systemic inflammation, and cirrhosis. J Hepatol. 2014;61:396-407.
50. Merlot AM, Kalinowski DS, Richardson DR. Unraveling the mysteries of serum albumin-more than just a serum protein. Front Physiol. 2014;5:299.
51. Dos Santos A, Court M, Thiers V, Sar S, Guettier C, Samuel D, et al. Identification of cellular targets in human intrahepatic cholangiocarcinoma using laser microdissection and accurate mass and time tag proteomics. Mol Cell Proteomics. 2010;9:1991-2004.
52. Waghray A, Sobotka A, Marrero CR, Estfan B, Aucejo F, Narayanan Menon KV. Serum albumin predicts survival in patients with hilar cholangiocarcinoma. Gastroenterol Rep (Oxf). 2017;5:62-6.
53. Garimella PS, Sarnak MJ. Uromodulin in kidney health and disease. Curr Opin Nephrol Hypertens. 2017;26:136-42.
54. Liu Y, El-Achkar TM, Wu XR. Tamm-Horsfall protein regulates circulating and renal cytokines by affecting glomerular filtration rate and acting as a urinary cytokine trap. J Biol Chem. 2012;287:16365-78.
55. Micanovic R, Chitteti BR, Dagher PC, Srour EF, Khan S, Hato T, et al. Tamm-Horsfall Protein Regulates Granulopoiesis and Systemic Neutrophil Homeostasis. J Am Soc Nephrol. 2015;26:2172-82.
56. El-Achkar TM, Wu XR, Rauchman M, McCracken R, Kiefer S, Dagher PC. Tamm-Horsfall protein protects the kidney from ischemic injury by decreasing inflammation and altering TLR4 expression. Am J Physiol Renal Physiol. 2008;295:F534-44.
57. Wu TH, Li KJ, Yu CL, Tsai CY. Tamm-Horsfall Protein is a Potent Immunomodulatory Molecule and a Disease Biomarker in the Urinary System. Molecules. 2018;23. pii: E200.
58. Heitmeier M, McCracken R, Micanovic R, Khan S, El-Achkar TM. The role of tumor necrosis factor alpha in regulating the expression of Tamm-Horsfall Protein (uromodulin) in thick ascending limbs during kidney injury. Am J Nephrol. 2014;40:458-67.
59. Sneha P, Thirumal Kumar D, Lijo J, Megha M, Siva R, George Priya Doss C. Probing the Protein-Protein Interaction Network of Proteins Causing Maturity Onset Diabetes of the Young. Adv Protein Chem Struct Biol. 2018;110:167-202.
60. Hiemstra TF, Charles PD, Hester SS, Karet FE, Lilley KS. Uromodulin exclusion list improves urinary exosomal protein identification. J Biomol Tech. 2011;22:136-45.
61. Bryan RT, Wei W, Shimwell NJ, Collins SI, Hussain SA, Billingham LJ, et al. Assessment of high-throughput high-resolution MALDI-TOF-MS of urinary peptides for the detection of muscle-invasive bladder cancer. Proteomics Clin Appl. 2011;5:493-503.
62. Abrahamson M, Barrett AJ, Salvesen G, Grubb A. Isolation of six cysteine proteinase inhibitors from human urine. Their physicochemical and enzyme kinetic properties and concentrations in biological fluids. J Biol Chem. 1986;261:11282-9.
63. Colman RW, Wu Y, Liu Y. Mechanisms by which cleaved kininogen inhibits endothelial cell differentiation and signalling. Thromb Haemost. 2010;104:875-85.
64. Weidmann H, Heikaus L, Long AT, Naudin C, Schlüter H, Renné T. The plasma contact system, a protease cascade at the nexus of inflammation, coagulation and immunity. Biochim Biophys Acta Mol Cell Res. 2017;1864:2118-27.
65. Wang J, Wang X, Lin S, Chen C, Wang C, Ma Q, Jiang B. Identification of kininogen-1 as a serum biomarker for the early detection of advanced colorectal adenoma and colorectal cancer. PLoS One. 2013;8:e70519.
66. Siebert S, Porter D, Paterson C, Hampson R, Gaya D, Latosinska A, et al. Urinary proteomics can define distinct diagnostic inflammatory arthritis subgroups. Sci Rep. 2017;7:40473.
67. Kistler AD, Serra AL, Siwy J, Poster D, Krauer F, Torres VE, et al. Urinary proteomic biomarkers for diagnosis and risk stratification of autosomal dominant polycystic kidney disease: a multicentric study. PLoS One. 2013;8(1):e53016.
68. Zhang ZY, Ravassa S, Nkuipou-Kenfack E, Yang WY, Kerr SM, Koeck T, et al. Novel Urinary Peptidomic Classifier Predicts Incident Heart Failure. J Am Heart Assoc. 2017;6: e005432.
69. Bixel MG, Li H, Petri B, Khandoga AG, Khandoga A, Zarbock A, et al. CD99 and CD99L2 act at the same site as, but independently of, PECAM-1 during leukocyte diapedesis. Blood. 2010;116:1172-84.
70. Nam G, Lee YK, Lee HY, Ma MJ, Araki M, Araki K, et al. Interaction of CD99 with its paralog CD99L2 positively regulates CD99L2 trafficking to cell surfaces. J Immunol. 2013;191:5730-42.
71. Song J, Zhang X, Buscher K, Wang Y, Wang H, Di Russo J, et al. Endothelial Basement Membrane Laminin 511 Contributes to Endothelial Junctional Tightness and Thereby Inhibits Leukocyte Transmigration. Cell Rep. 2017;18:1256-69.
72. Huang RY, Kuay KT, Tan TZ, Asad M, Tang HM, Ng AH, et al. Functional relevance of a six mesenchymal gene signature in epithelial-mesenchymal transition (EMT) reversal by the triple angiokinase inhibitor, nintedanib (BIBF1120). Oncotarget. 2015;6:22098-113.
73. Di Meo A, Batruch I, Yousef AG, Pasic MD, Diamandis EP, Yousef GM. An integrated proteomic and peptidomic assessment of the normal human urinome. Clin Chem Lab Med. 2017;55:237-47.
74. Somasundaram R, Schuppan D. Type I, II, III, IV, V, and VI collagens serve as extracellular ligands for the isoforms of platelet-derived growth factor (AA, BB, and AB). J Biol Chem. 1996;271:26884-91.
75. Liu J, Shen JX, Wu HT, Li XL, Wen XF, Du CW, et al. Collagen 1A1 (COL1A1) promotes metastasis of breast cancer and is a potential therapeutic target. Discov Med 2018;25:211-23.
76. Liu S, Liao G, Li G. Regulatory effects of COL1A1 on apoptosis induced by radiation in cervical cancer cells. Cancer Cell Int. 2017;17:73.
77. Zhang XF, Dong M, Pan YH, Chen JN, Huang XQ, Jin Y, Shao CK. Expression pattern of cancer-associated fibroblast and its clinical relevance in intrahepatic cholangiocarcinoma. Hum Pathol. 2017;65:92-100.
78. Schanstra JP, Zürbig P, Alkhalaf A, Argiles A, Bakker SJ, Beige J, et al. Diagnosis and Prediction of CKD Progression by Assessment of Urinary Peptides. J Am Soc Nephrol. 2015;26:1999-2010.
79. Wei R, Gao B, Shih F, Ranger A, Dearth A, Mischak H, et al. Alterations in urinary collagen peptides in lupus nephritis subjects correlate with renal dysfunction and renal histopathology. Nephrol Dial Transplant. 2017;32:1468-77.
80. Yasuda H, Oh CD, Chen D, de Crombrugghe B, Kim JH. A Novel Regulatory Mechanism of Type II Collagen Expression via a SOX9-dependent Enhancer in Intron 6. J Biol Chem. 2017;292:528-38.

81. Flowers SA, Zieba A, Örnros J, Jin C, Rolfson O, Björkman LI, et al. Lubricin binds cartilage proteins, cartilage oligomeric matrix protein, fibronectin and collagen II at the cartilage surface. Sci Rep. 2017;7:13149.
82. Ganapathi MK, Jones WD, Sehouli J, Michener CM, Braicu IE, Norris EJ, et al. Expression profile of COL2A1 and the pseudogene SLC6A10P predicts tumor recurrence in high-grade serous ovarian cancer. Int J Cancer. 2016;138:679-88.
83. Murakawa K, Tada M, Takada M, Tamoto E, Shindoh G, Teramoto K, et al. Prediction of lymph node metastasis and perineural invasion of biliary tract cancer by selected features from cDNA array data. J Surg Res. 2004;122:184-94.
84. Nowack H, Gay S, Wick G, Becker U, Timpl R. Preparation and use in immunohistology of antibodies specific for type I and type III collagen and procollagen. J Immunol Methods. 1976;12:117-24.
85. Sixma JJ, van Zanten GH, Huizinga EG, van der Plas RM, Verkley M, Wu YP, et al. Platelet adhesion to collagen: an update. Thromb Haemost. 1997;78:434-8.
86. Yuan L, Shu B, Chen L, Qian K, Wang Y, Qian G, et al. Overexpression of COL3A1 confers a poor prognosis in human bladder cancer identified by co-expression analysis. Oncotarget. 2017;8:70508-20.
87. Rong L, Huang W, Tian S, Chi X, Zhao P, Liu F. COL1A2 is a Novel Biomarker to Improve Clinical Prediction in Human Gastric Cancer: Integrating Bioinformatics and Meta-Analysis. Pathol Oncol Res. 2018;24:129-34.
88. Lai KK, Shang S, Lohia N, Booth GC, Masse DJ, Fausto N, et al. Extracellular matrix dynamics in hepatocarcinogenesis: a comparative proteomics study of PDGFC transgenic and Pten null mouse models. PLoS Genet. 2011;7:e1002147.
89. Good DM, Zürbig P, Argiles A, Bauer HW, Behrens G, Coon JJ, et al. Naturally occurring human urinary peptides for use in diagnosis of chronic kidney disease. Mol Cell Proteomics 2010;9:2424-37.
90. Meng XY, Shi MJ, Zeng ZH, Chen C, Liu TZ, Wu QJ, Li S, Li S. The Role of COL5A2 in Patients with Muscle-Invasive Bladder Cancer: A Bioinformatics Analysis of Public Datasets Involving 787 Subjects and 29 Cell Lines. Front Oncol. 2019 Jan 15;8:659.
91. Groulx JF, Gagné D, Benoit YD, Martel D, Basora N, Beaulieu JF. Collagen VI is a basement membrane component that regulates epithelial cell-fibronectin interactions. Matrix Biol. 2011;30:195-206.
92. Yeh MH, Tzeng YJ, Fu TY, You JJ, Chang HT, Ger LP, Tsai KW. Extracellular Matrix-receptor Interaction Signaling Genes Associated with Inferior Breast Cancer Survival. Anticancer Res. 2018;38:4593-605.
93. Koster J, Geerts D, Favre B, Borradori L, Sonnenberg A. Analysis of the interactions between BP180, BP230, plectin and the integrin alpha6beta4 important for hemidesmosome assembly. J Cell Sci. 2003;116:387-99.
94. Thangavelu PU, Krenács T, Dray E, Duijf PH. In epithelial cancers, aberrant COL17A1 promoter methylation predicts its misexpression and increased invasion. Clin Epigenetics. 2016;8:120.
95. Chapman MH, Tidswell R, Dooley JS, Sandanayake NS, Cerec V, Deheragoda M, et al. Whole genome RNA expression profiling of endoscopic biliary brushings provides data suitable for biomarker discovery in cholangiocarcinoma. J Hepatol. 2012;56:877-85.
96. Therien AG, Karlish SJ, Blostein R. Expression and functional role of the gamma subunit of the Na, K-ATPase in mammalian cells. J Biol Chem. 1999;274:12252-6.
97. Hsu IL, Chou CY, Wu YY, Wu JE, Liang CH, Tsai YT, et al. Targeting FXYD2 by cardiac glycosides potently blocks tumor growth in ovarian clear cell carcinoma. Oncotarget. 2016;7:62925-38.
98. Li Y, Zhang X, Xu S, Ge J, Liu J, Li L, et al. Expression and clinical significance of FXYD3 in endometrial cancer. Oncol Lett. 2014;8:517-22.
99. Gu YM, Thijs L, Liu YP, Zhang Z, Jacobs L, Koeck T, et al. The urinary proteome as correlate and predictor of renal function in a population study. Nephrol Dial Transplant. 2014;29:2260-8.
100. Ryu CS, Klein K, Zanger UM. Membrane Associated Progesterone Receptors: Promiscuous Proteins with Pleiotropic Functions - Focus on Interactions with Cytochromes P450. Front Pharmacol. 2017;8:159.
101. Kaluka D, Batabyal D, Chiang BY, Poulos TL, Yeh SR. Spectroscopic and mutagenesis studies of human PGRMC1. Biochemistry. 2015;54:1638-47.
102. Kabe Y, Nakane T, Koike I, Yamamoto T, Sugiura Y, Harada E, et al. Haem-dependent dimerization of PGRMC1/Sigma-2 receptor facilitates cancer proliferation and chemoresistance. Nat Commun. 2016;7:11030.
103. Latosinska A, Mokou M, Makridakis M, Mullen W, Zoidakis J, Lygirou V, et al. Proteomics analysis of bladder cancer invasion: Targeting EIF3D for therapeutic intervention. Oncotarget. 2017 Apr;8:69435-55.
104. Zhang Y, Ruan X, Willibald M, Seeger H, Fehm T, Neubauer H, Mueck AO. May progesterone receptor membrane component 1 (PGRMC1) predict the risk of breast cancer? Gynecol Endocrinol. 2016;32:58-60.
105. Tsai HW, Ho CL, Cheng SW, Lin YJ, Chen CC, Cheng PN, et al. Progesterone receptor membrane component 1 as a potential prognostic biomarker for hepatocellular carcinoma. World J Gastroenterol. 2018;24:1152-66.
106. Rossing K, Bosselmann HS, Gustafsson F, Zhang ZY, Gu YM, Kuznetsova T, et al. Urinary Proteomics Pilot Study for Biomarker Discovery and Diagnosis in Heart Failure with Reduced Ejection Fraction. PLoS One. 2016;11:e0157167.
107. Schiffer E, Vlahou A, Petrolekas A, Stravodimos K, Tauber R, Geschwend JE, et al. Prediction of muscle-invasive bladder cancer using urinary proteomics. Clin Cancer Res. 2009;15:4935-43.
108. Guttman O, Baranovski BM, Schuster R, Kaner Z, Freixo-Lima GS, Bahar N, et al. Acute-phase protein α1-anti-trypsin: diverting injurious innate and adaptive immune responses from non-authentic threats. Clin Exp Immunol. 2015;179(2):161-72.
109. Janciauskiene SM, Bals R, Koczulla R, Vogelmeier C, Köhnlein T, Welte T. The discovery of α1-antitrypsin and its role in health and disease. Respir Med. 2011;105:1129-39.
110. Abecassis A, Schuster R, Shahaf G, Ozeri E, Green R, Ochayon DE, et al. α1-antitrypsin increases interleukin-1 receptor antagonist production during pancreatic islet graft transplantation. Cell Mol Immunol. 2014;11:377-86.
111. Haddad A, Kowdley GC, Pawlik TM, Cunningham SC. Hereditary pancreatic and hepatobiliary cancers. Int J Surg Oncol. 2011;2011:154673.
112. Dahl M, Tybjaerg-Hansen A, Lange P, Vestbo J, Nordestgaard BG. Change in lung function and morbidity from chronic obstructive pulmonary disease in alpha1-antitrypsin MZ heterozygotes: A longitudinal study of the general population. Ann Intern Med. 2002;136:270-9.
113. Mota A, Sahebghadam Lotfi A, Jamshidi AR, Najavand S. Alpha 1-antitrypsin activity is markedly decreased in Wegener's granulomatosis. Rheumatol Int. 2014;34:553-8.
114. Sriwanitchrak P, Viyanant V, Chaijaroenkul W, Srivatanakul P, Gram HR, Eursiddhichai V, Na-Bangchang K. Proteomics analysis and evaluation of biomarkers for detection of cholangiocarcinoma. Asian Pac J Cancer Prev. 2011;12:1503-10.
115. Jamnongkan W, Techasen A, Thanan R, Duenngai K, Sithithaworn P, Mairiang E, et al. Oxidized alpha-1 antitrypsin as a predictive risk marker of opisthorchiasis-associated cholangiocarcinoma. Tumour Biol. 2013;34:695-704.
116. Cherney D, Perkins BA, Lytvyn Y, Heerspink H, Rodríguez-Ortiz ME, Mischak H. The effect of sodium/glucose cotransporter 2 (SGLT2) inhibition on the urinary proteome. PLoS One. 2017;12:e0186910.
117. Radons J, Dove S, Neumann D, Altmann R, Botzki A, Martin MU, Falk W. The interleukin 1 (IL-1) receptor accessory protein Toll/IL-1 receptor domain: analysis of putative interaction sites in vitro mutagenesis and molecular modeling. J Biol Chem. 2003;278:49145-53.
118. Mora J, Schlemmer A, Wittig I, Richter F, Putyrski M, Frank AC, et al. Interleukin-38 is released from apoptotic cells to limit inflammatory macrophage responses. J Mol Cell Biol. 2016;8:426-38.
119. Bahi N, Friocourt G, Carrié A, Graham ME, Weiss JL, Chafey P, et al. IL1 receptor accessory protein like, a protein involved in X-linked mental retardation, interacts with Neuronal Calcium Sensor-1 and regulates exocytosis. Hum Mol Genet. 2003;12(12):1415-25.
120. Khan JA, Brint EK, O'Neill LA, Tong L. Crystal structure of the Toll/interleukin-1 receptor domain of human IL-1RAPL. J Biol Chem. 2004;279:31664-70.
121. Lai RC, Yeo RWY, Tan SS, Zhang B, Yin Y, Sze NSK, et al. Mesenchymal Stem Cell Exosomes: The Future MSC-Based Therapy?. In: Chase L, Vemuri M, editors. Mesenchymal Stem Cell Therapy. Stem Cell Biology and Regenerative Medicine. Totowa, NJ: Humana Press; 2013. p. 39-61.
122. Han Y, Mora J, Putyrski M, Huard A, da Silva P, Scholz A, et al. Il-38 Restricts Skin Inflammation and Anti-Tumor Immunity by Limiting Il-17 Production from γδ T Cells. *Cell Reports*. 2018; doi:10.2139/ssrn.3213912.
123. Gao L, Yang X, Zhang H, Yu M, Long J, Yang T. Inhibition of miR-10a-5p suppresses cholangiocarcinoma cell growth through downregulation of Akt pathway. Onco Targets Ther. 2018;11:6981-94.

124. Pettersen RD, Bernard G, Olafsen MK, Pourtein M, Lie SO. CD99 signals caspase-independent T cell death. J Immunol. 2001;166:4931-42.
125. Lee KJ, Lee SH, Yadav BK, Ju HM, Kim MS, Park JH, et al. The activation of CD99 inhibits cell-extracellular matrix adhesion by suppressing β(1) integrin affinity. BMB Rep. 2012;45:159-64.
126. Edlund K, Lindskog C, Saito A, Berglund A, Pontén F, Göransson-Kultima H, et al. CD99 is a novel prognostic stromal marker in non-small cell lung cancer. Int J Cancer. 2012;131:2264-73.
127. Kovar H, Dworzak M, Strehl S, Schnell E, Ambros IM, Ambros PF, Gadner H. Overexpression of the pseudoautosomal gene MIC2 in Ewing's sarcoma and peripheral primitive neuroectodermal tumor. Oncogene. 1990;5:1067-70.
128. Zhou G, Yang W, Yu L, Yu T, Liu Z. CD99 refers to the activity of inflammatory bowel disease. Scand J Gastroenterol. 2017;52:359-64.
129. Choi YL, Xuan YH, Shin YK, Chae SW, Kook MC, Sung RH, et al. An immunohistochemical study of the expression of adhesion molecules in gallbladder lesions. J Histochem Cytochem. 2004;52:591-601.
130. Lee JH, Kim SH, Wang LH, Choi YL, Kim YC, Kim JH, et al. Clinical significance of CD99 down-regulation in gastric adenocarcinoma. Clin Cancer Res. 2007;13:2584-91.
131. Zurick KM, Qin C, Bernards MT. Mineralization induction effects of osteopontin, bone sialoprotein, and dentin phosphoprotein on a biomimetic collagen substrate. J Biomed Mater Res A. 2013;101:1571-81.
132. Sodek J, Zhu B, Huynh MH, Brown TJ, Ringuette M. Novel functions of the matricellular proteins osteopontin and osteonectin/SPARC. Connect Tissue Res. 2002;43:308-19.
133. Zhu B, Suzuki K, Goldberg HA, Rittling SR, Denhardt DT, McCulloch CA, Sodek J. Osteopontin modulates CD44-dependent chemotaxis of peritoneal macrophages through G-protein-coupled receptors: evidence of a role for an intracellular form of osteopontin. J Cell Physiol. 2004;198:155-67.
134. Ashkar S, Weber GF, Panoutsakopoulou V, Sanchirico ME, Jansson M, Zawaideh S, et al. Eta-1 (osteopontin): an early component of type-1 (cell-mediated) immunity. Science. 2000;287:860-4.
135. Rangaswami H, Bulbule A, Kundu GC. Osteopontin: role in cell signaling and cancer progression. Trends Cell Biol. 2006;16:79-87.
136. Wang X, Lopategi A, Ge X, Lu Y, Kitamura N, Urtasun R, et al. Osteopontin induces ductular reaction contributing to liver fibrosis. Gut. 2014;63:1805-18.
137. Klement JD, Paschall AV, Redd PS, Ibrahim ML, Lu C, Yang D, et al. An osteopontin/CD44 immune checkpoint controls CD8+ T cell activation and tumor immune evasion. J Clin Invest. 2018;128:5549-60.
138. Shurin MR. Osteopontin controls immunosuppression in the tumor microenvironment. J Clin Invest. 2018;128:5209-12.
139. Loosen SH, Roderburg C, Kauertz KL, Pombeiro I, Leyh C, Benz F, et al. Elevated levels of circulating osteopontin are associated with a poor survival after resection of cholangiocarcinoma. J Hepatol. 2017;67:749-57.
140. Zheng Y, Zhou C, Yu XX, Wu C, Jia HL, Gao XM, et al. Osteopontin promotes metastasis of intrahepatic cholangiocarcinoma through recruiting MAPK1 and mediating Ser675 phosphorylation of β-Catenin. Cell Death Dis. 2018;9:179.
